# Deacylated tRNA Accumulation Is a Trigger for Bacterial Antibiotic Persistence Independent of the Stringent Response

**DOI:** 10.1128/mBio.01132-21

**Published:** 2021-06-15

**Authors:** Whitney N. Wood, Kyle Mohler, Jesse Rinehart, Michael Ibba

**Affiliations:** a Department of Microbiology, The Ohio State University, Columbus, Ohio, USA; b Department of Cellular & Molecular Physiology, Yale School of Medicine, New Haven, Connecticut, USA; c Systems Biology Institute, Yale University, New Haven, Connecticut, USA; d Schmid College of Science and Technology, Chapman University, Orange, California, USA; Vanderbilt University

**Keywords:** persistence, tRNA, translation

## Abstract

Bacterial antibiotic persistence occurs when bacteria are treated with an antibiotic and the majority of the population rapidly dies off, but a small subpopulation enters into a dormant, persistent state and evades death. Diverse pathways leading to nucleoside triphosphate (NTP) depletion and restricted translation have been implicated in persistence, suggesting alternative redundant routes may exist to initiate persister formation. To investigate the molecular mechanism of one such pathway, functional variants of an essential component of translation (phenylalanyl-tRNA synthetase [PheRS]) were used to study the effects of quality control on antibiotic persistence. Upon amino acid limitation, elevated PheRS quality control led to significant decreases in aminoacylated tRNA^Phe^ accumulation and increased antibiotic persistence. This increase in antibiotic persistence was most pronounced (65-fold higher) when the *relA-*encoded tRNA-dependent stringent response was inactivated. The increase in persistence with elevated quality control correlated with ∼2-fold increases in the levels of the RNase MazF and the NTPase MazG and a 3-fold reduction in cellular NTP pools. These data reveal a mechanism for persister formation independent of the stringent response where reduced translation capacity, as indicated by reduced levels of aminoacylated tRNA, is accompanied by active reduction of cellular NTP pools which in turn triggers antibiotic persistence.

## INTRODUCTION

Bacterial persisters are a subpopulation of dormant microbial cells that are not actively growing or dividing, which causes them to be tolerant to bactericidal antibiotics (1). Once the antibiotic reaches a low-enough concentration, persisters are able to resuscitate and the bacterial population resumes growth, leading to infection relapse and chronic bacterial infections (2). This mechanism of antibiotic-induced persisters is an example of triggered persistence, one of two different types of bacterial persistence. Different triggers that can cause bacterial cells to enter into a persistent state include antibiotics, amino acid starvation, immune factors, and other physiological stressors. Another type of persistence is spontaneous, which occurs when a subpopulation of bacteria becomes dormant while the vast majority of the bacterial culture are in the exponential phase of growth (3). Persistence can be characterized by the observation that the majority of a bacterial culture will be rapidly killed by exposure to an antibiotic while the subpopulation of persisters will have a much lower rate of killing. These different responses to antibiotics by the persistent and nonpersistent cells result in a biphasic killing curve that can be quantified by the minimum duration of killing (4). Antibiotic persistence differs from both resistance and tolerance. Antibiotic resistance is when bacteria are able to actively grow in the presence of antibiotics demonstrated by their elevated MIC for that antibiotic. Although antibiotic tolerance is similar to antibiotic persistence, the main difference is that tolerance affects the entire population resulting in a lower killing rate than with nontolerant bacteria but without any accompanying change in MIC (3, 4).

The health risk that persisters pose has caused them to receive considerable attention; however, the mechanisms underlying how persisters form remain elusive (5). Since persisters make up a small subpopulation of cells that have low biochemical activity, studying them presents challenges. Much of our current understanding of persisters comes from selection of high-persister mutants, those that display elevated levels of antibiotic tolerance, and from screenings for persistence using knockout libraries and transcriptome analyses (6–9). Several genes have been identified that might contribute to the dormant persister phenotype, many of which are members of toxin/antitoxin (TA) module systems. These modules are comprised of a stable toxin protein that when bound to the antitoxin forms an inactive complex; in the absence of the antitoxin, critical cellular functions such as protein translation or DNA replication are inhibited and cell growth is halted (1). One pathway to persister formation, via a TA module, involves the alarmone ppGpp. Several different external stressors can induce the synthesis of ppGpp, which activates Lon protease. The Lon protease degrades the antitoxin leading to inhibition of translation, slowing down cellular growth and increasing antibiotic tolerance (10, 11). However, this model for persister formation has recently been challenged, when it was shown that persister formation still occurs in an Escherichia coli Δ*relA* Δ*spoT* background that is unable to synthesize ppGpp (12, 13). Additionally, another study has recently questioned the role of TA modules in persister formation (14). This new model proposes that a drop in intracellular ATP concentration acts as a trigger for persistence. This hypothesis proposes that a low concentration of ATP in the cell leads to a decrease in the activities of key antibiotic targets such as protein synthesis, DNA replication, cell wall biosynthesis, etc., leading to persistence (9, 12, 15). These results suggest that there is not a single route to persister formation; rather, there may exist several redundant pathways, all of which can lead to persistence. However, the identities of these pathways, their roles, and their possible connections to each other remain largely unclear.

Recently, several studies showed that perturbations in the protein synthesis machinery have effects on bacterial persistence (16–22). Two of these studies involved the toxin HipA, which can phosphorylate glutamyl-tRNA synthetase (GluRS), thereby inactivating it. Aminoacyl-tRNA synthetases (aaRS) esterify amino acids to their cognate tRNA, and the resulting aminoacyl-tRNA (aa-tRNA) forms a ternary complex with EF-Tu and GTP which is then used as a substrate for ribosomal protein synthesis (23). When GluRS is inactivated by HipA, it is unable to synthesize Glu-tRNA^Glu^, and the intracellular concentration of deacylated tRNA^Glu^ increases. Deacylated tRNA^Glu^ directly enters the A-site of the ribosome which triggers the activation of RelA, a ppGpp synthase, and the stringent response is turned on, which causes RelA-dependent bacterial persistence (16). It was recently shown that HipA can also phosphorylate TrpRS and LysRS in addition to GluRS (21). Mutations in the genes encoding IleRS, LeuRS, ProRS, and MetRS have also been found that caused an increase in bacterial persistence. These mutations were identified in a ΔTA11 Escherichia coli strain, which has all 10 type II TA modules and the *hipBA* locus deleted, indicating that these persisters form via a different molecular mechanism than the one described previously where a toxin led to translation inhibition (19). Antibiotics that target TrpRS and LeuRS have also been used to study persistence in Chlamydia, which does not have the genes that encode RelA or SpoT and so does not appear to utilize the stringent response (20, 24). The Chlamydia persisters form by a molecular mechanism similar to the one described previously in which their trigger for persistence seems to be a direct effect from slowing down translation via accumulation of deacylated tRNA but independent of the stringent response.

Several studies have shown that the accuracy and efficiency of aminoacyl-tRNA synthesis are critical determinants of bacterial homeostasis (25–27). To investigate how this global role of aaRSs might impact persistence, mutations in *pheS* and *pheT*, which encode the α-subunit and β-subunits of PheRS, respectively, were investigated in both E. coli MG1655 *relA*^+^ and Δ*relA* backgrounds. Changes were made in both the active site and editing sites of PheRS to investigate the effects of varying aminoacylation efficiency and accuracy, respectively. The PheRS editing site is used to clear misacylated Tyr-tRNA^Phe^ and *m*-Tyr-tRNA^Phe^. *m*-Tyr is a product of Phe oxidation and, in the absence of PheRS editing, has been shown to be mistranslated, causing cytotoxicity and other growth defects in E. coli (28, 29). PheRS quality control is also important beyond its primary role in maintaining translation accuracy, as demonstrated by the finding that when *m*-Tyr-tRNA^Phe^ is not hydrolyzed by PheRS, the stringent response is suppressed (25). Changes in the active site of PheRS led to a significant decrease in the amount of aminoacylated tRNA^Phe^ in response to amino acid stress, and this also resulted in a significant increase in antibiotic persistence but only in the strain that is unable to mount the stringent response. These data indicate that disruption of bacterial homeostasis via both reduced translation quality control and suppression of the stringent response together increases antibiotic persistence.

## RESULTS

### Changes in the active site of PheRS perturb discrimination against noncognate amino acids.

It has been previously shown that an editing-deficient PheRS E. coli variant, *pheT* G318W, had significant effects on cellular homeostasis under oxidative and amino acid stress (25, 29). This strain is not able to edit Tyr-tRNA^Phe^ or *m*-Tyr-tRNA^Phe^, and consequently *m*-Tyr, a nonproteogenic amino acid, was shown to be incorporated into the proteome and cause cytotoxicity (28, 29). This PheRS editing-deficient strain was also shown to not activate the stringent response upon amino acid stress by *m*-Tyr addition (25). Taken together, these previous findings provided a basis to investigate the effects of PheRS quality control on bacterial antibiotic persistence and its dependence on the stringent response.

Along with an E. coli PheRS editing-deficient strain, additional E. coli mutant strains were made which chromosomally encode PheRS active site variants in both MG1655 *relA*^+^ and Δ*relA* backgrounds (29–31). The two active site mutations that were made on the E. coli chromosome encode the A294G and A294S variants in the α-subunit of PheRS. The A294G replacement in the active site of PheRS is predicted to have reduced amino acid substrate discrimination while the A294S replacement is predicted to have increased discrimination (32). These replacements were also made in recombinant E. coli PheRS to allow determination of the amino acid activation kinetics for the PheRS active site variants (Table 1). The specificity of Phe-to-*m*-Tyr for wild-type (WT) PheRS is 22, and for αA294G PheRS, it is 1.8, confirming that editing is required to prevent misacylated *m*-Tyr-tRNA^Phe^ accumulation and that αA294G PheRS can only minimally discriminate between Phe and *m*-Tyr. In contrast, the specificity of Phe-to-*m*-Tyr for αA294S PheRS is 370, indicating that this mutant is not able to efficiently activate *m*-Tyr for aminoacylation. The same trend follows for the specificity of Phe-to-Tyr: αA294G PheRS has a reduced substrate specificity compared to wild-type PheRS, and αA294S PheRS has an increased specificity.

**TABLE 1:** Steady-state kinetics of amino acid activation by E. coli wild-type PheRS and two active site PheRS mutants

PheRS	Phe			*m*-Tyr			Tyr^*a*^	Specificity	
	*K_m_* (μM)	*k*_cat_ (s^−1^)	*k*_cat_/*K_m_* (s^−1^/μM)	*K_m_* (μM)	*k*_cat_ (s^−1^)	*k*_cat_/*K_m_* (s^−1^/μM)	*k*_cat_/*K_m_* (s^−1^/μM)	Phe/*m*-Tyr	Phe/Tyr
WT	21 ± 1	61 ± 11	2.9	175 ± 31	23 ± 3	0.13	0.002 ± 0.0004	22	1,400
αA294G	59 ± 29	58 ± 16	1.0	62 ± 7	36 ± 6	0.6	0.01 ± 0.006	1.8	110
αA294S	27 ± 7	97 ± 10	3.6	ND^*a*^	ND^*a*^	0.01 ± 0.003	0.001 ± 0.0002	370	3,700

a Individual kinetic parameters could not be determined due to a high *K_m_* and substrate solubility. *k*_cat_/*K_m_* was estimated by ν = *k*_cat_/*K_m_* ([E][S]). Standard deviation is from 3 replicates. ND, not determined.

### Bacterial antibiotic persistence increases when quality control is present, and the stringent response is disrupted.

**FIG 1:**
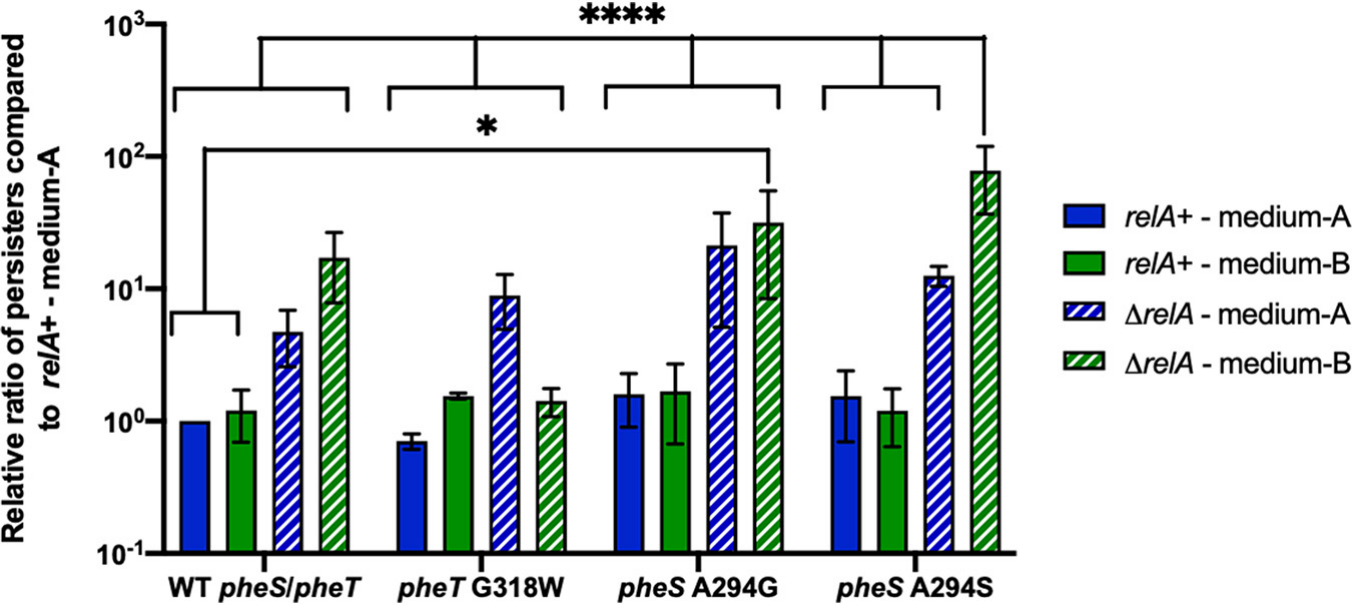
Antibiotic persistence increases when quality control is present, and the stringent response is disrupted. Persistence assays were done with wild-type *pheS/pheT*, *pheT* G318W, *pheS* A294G, and *pheS* A294S in both E. coli MG1655 *relA*^+^ (solid bars) and a XΔ*relA* -36292-8262-3643 strain (striped bars). Persistence was measured and quantified after 3 h of exposure to 100 μg/ml ampicillin and is shown on a log scale set relative to wild-type *pheS/pheT* in the *relA*^+^ background grown in medium A. Medium A (blue) is a supplemented M9 minimal medium that contains 40 μg/ml of all 20 proteogenic amino acids, and medium B (green) is a supplemented M9 minimal medium with 40 μg/ml of 18 proteogenic amino acids, 10 μg/ml Tyr, 40 μg/ml *m*-Tyr, and no Phe. Error bars represent standard deviations from 3 biological replicates. WT *pheS/pheT relA*^+^ in medium A and medium B is significant to *pheS* A294G Δ*relA* in medium B with a *P* value of <0.03. All data sets are significant to *pheS* A294S Δ*relA* in medium B with a *P* value of <0.0001, except for *pheS* A294G Δ*relA* in medium B with a *P* value of 0.0008. Statistical analysis was performed using two-way analysis of variance (ANOVA).

To investigate if differences in noncognate substrate discrimination by PheRS variants affect antibiotic persistence, minimum duration of killing (MDK) assays were performed in both MG1655 *relA*^+^ and Δ*relA* E. coli backgrounds (see Fig. S1 in the supplemental material). The four different PheRS strains (wild-type *pheS/pheT*, *pheT* G318W, *pheS* A294G, and *pheS* A294S) in both backgrounds were grown to early log phase and then treated with 100 μg/ml ampicillin for 3 h; each hour, an aliquot was taken out, washed with sterile phosphate-buffered saline (PBS), plated on LB plates, and incubated at 37°C overnight to calculate CFU. These assays were performed in two different types of media. The first, medium A, is the control medium which is a supplemented M9-based minimal medium that contains 40 μg/ml of all 20 proteogenic amino acids. The second, medium B, is the starvation medium which is also a supplemented M9-based minimal medium that contains 40 μg/ml of 18 proteogenic amino acids, 10 μg/ml Tyr, 40 μg/ml *m*-Tyr, and no Phe. The total amounts of persisters for wild-type PheRS, editing-deficient PheRS, and the two different active site PheRS mutant strains in both E. coli backgrounds were calculated at the endpoint after 3 h of exposure to ampicillin (Fig. 1). There was a significant increase in persisters for *pheS* A294S in the Δ*relA* background grown in medium B compared to all the other PheRS strains that were tested. Three different variables were tested in this assay: *relA* either present or knocked out, different *pheT* and *pheS* mutations, and nutrient limitations. Each of the variables had an independent effect on persistence. For example, the deletion of *relA* caused an increase in persistence when the other two variables remained constant (compare solid blue bar and striped blue bar in WT *pheS*/*pheT* data set in Fig. 1). When the three different variables are combined, deletion of *relA*, PheRS quality control, and amino acid starvation, the effect on persistence is compounded and the largest amount of persister cell formation is observed. These data indicate that this pathway for bacterial antibiotic persistence is independent of the RelA-dependent stringent response.

10.1128/mBio.01132-21.2 FIG S1 Minimum duration of killing (MDK) assays for different E. coli PheRS strains in both a *relA*^+^ and Δ*relA* background. Cultures were grown to early log phase and then exposed to 100 μg/ml ampicillin for 3 h. Cultures were taken out every hour, washed in sterile PBS, and plated to calculate CFU. (A) WT *pheS*/*pheT*; (B) editing-deficient PheRS, *pheT* G318W; (C) active site mutant PheRS, *pheS* A294G; (D) active site mutant *pheS* A294S. In each panel, MDK assay was performed in *relA*^+^ E. coli (black lines), Δ*relA* E. coli (colored lines), medium A (solid lines), and medium B (dashed lines). Medium A is a supplemented M9 minimal medium that contains 40 μg/ml of all 20 proteogenic amino acids, and medium B is a supplemented M9 minimal medium with 40 μg/ml of 18 proteogenic amino acids, 10 μg/ml Tyr, 40 μg/ml *m*-Tyr, and no Phe. Error bars represent standard deviation from 3 biological replicates. Download FIG S1, PDF file, 0.1 MB. Copyright © 2021 Wood et al. 2021 Wood et al. https://creativecommons.org/licenses/by/4.0/ This content is distributed under the terms of the Creative Commons Attribution 4.0 International license.

A *pheT* G318W/*pheS* A294G double mutant was made in both the E. coli MG1655 *relA*^+^ and Δ*relA* backgrounds. These strains would be able to synthesize, but not edit, *m*-Tyr-tRNA^Phe^ and Tyr-tRNA^Phe^ and therefore would generate high levels of misacylated tRNA^Phe^ in the cell. The *pheT* G318W/*pheS* A294G double mutation allowed normal growth compared to the wild-type PheRS in medium A in both the *relA*^+^ and Δ*relA* backgrounds. However, we were not able to perform persister assays on the *pheT* G318W/*pheS* A294G/Δ*relA* mutant because the cells showed a substantial growth defect in medium B (Fig. S2).

10.1128/mBio.01132-21.3 FIG S2 Growth curves comparing wild-type PheRS and double mutant *pheT* G318W/*pheS* A294G in both a *relA*^+^ and Δ*relA* background. Cultures were growing in either medium A or medium B, and OD_600_ was taken every hour for 8 h. Growth curves were performed with MG1655 E. coli *relA*^+^ with wild-type PheRS (solid line) and double mutant PheRS, *pheS* A294G/*pheT* G318W (dashed lines), in either medium A (green) or medium B (blue) or with Δ*relA* E. coli with wild-type PheRS (solid line) and double mutant PheRS (dashed lines) in either medium A (purple) or medium B (orange). Medium A is a supplemented M9 minimal medium that contains 40 μg/ml of all 20 proteogenic amino acids, and medium B is a supplemented M9 minimal medium with 40 μg/ml of 18 proteogenic amino acids, 10 μg/ml Tyr, 40 μg/ml *m*-Tyr, and no Phe. Error bars represent standard deviation from 3 biological replicates. Download FIG S2, PDF file, 0.1 MB. Copyright © 2021 Wood et al. 2021 Wood et al. https://creativecommons.org/licenses/by/4.0/ This content is distributed under the terms of the Creative Commons Attribution 4.0 International license.

### Levels of deacylated tRNA^Phe^ correlate with the levels of persisters for the active site mutants of PheRS.

**FIG 2:**
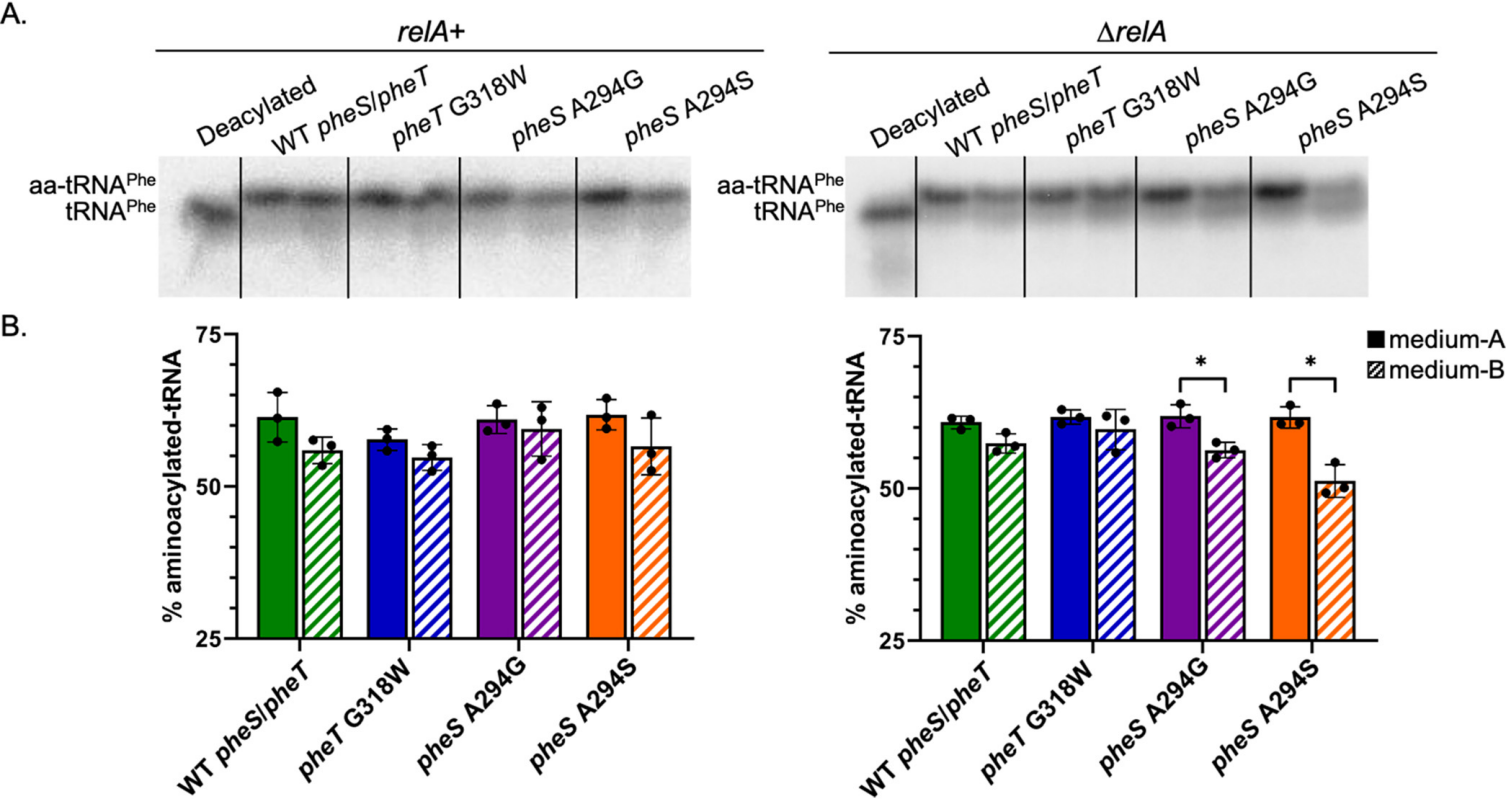
Levels of aminoacylated tRNA^Phe^ correlate with antibiotic persistence. (A) Representative Northern blots with a ^32^P-5′-end-labeled tRNA^Phe^ probe in which 10 μg total tRNA was separated by aminoacylated and deacylated tRNA species on an acid urea gel. For each strain, the left lane was grown in medium A and the right lane was grown in medium B. Left panel is tRNA purified from the E. coli MG1655 *relA*^+^ strain, and right panel is tRNA purified from the E. coli *ΔrelA* strain. (B) Percent aminoacylated tRNA^Phe^ levels *in vivo* in wild-type *pheS/pheT*, *pheT* G318W, *pheS* A294G, and *pheS* A294S in both the *relA*^+^ strain (left panel) and a Δ*relA* strain (right panel). Cultures were grown to late log phase in either medium A (solid bars), which is a supplemented M9 minimal medium that contains 40 μg/ml of all 20 proteogenic amino acids, or medium B (striped bars), which is a supplemented M9 minimal medium with 40 μg/ml of 18 proteogenic amino acids, 10 μg/ml Tyr, 40 μg/ml *m*-Tyr, and no Phe. Error bars represent standard deviations from 3 biological replicates. *, *P* value < 0.04; statistical analysis was performed using multiple *t* tests.

To investigate if there is a correlation between the levels of persistence and the levels of deacylated tRNA^Phe^, assays were performed to quantify tRNA^Phe^ aminoacylation in all four PheRS strains in both *relA*^+^ and Δ*relA* E. coli backgrounds grown in either medium A or medium B (Fig. 2). A representative Northern blot that was probed with a ^32^P-5′-end-labeled oligonucleotide that is specific for tRNA^Phe^ is shown (Fig. 2A), and quantification from biological triplicates was performed (Fig. 2B). Overall, there was no difference in the levels of aminoacylated-tRNA^Phe^ across all 4 different PheRS strains in the *relA*^+^ background grown in either medium A or medium B. The only instances in which significantly decreased levels of aminoacylated-tRNA^Phe^ were observed were for the Δ*relA* *pheS* A294G and Δ*relA* *pheS* A294S mutant strains grown in medium B, the same conditions that also gave rise to the highest observed levels of persistence in this study.

### Intracellular nucleotide concentrations indicate a reduction in metabolic activity when persistence increases.

The intracellular nucleotide concentrations were determined by letting the wild-type *pheS*/*pheT* and all three mutants in both the *relA*^+^ and Δ*relA* backgrounds grow in medium A and medium B to early log phase. Metabolites were extracted and then analyzed by liquid chromatography-mass spectrometry (LC-MS). There was a general trend of having a lower intracellular nucleotide concentration in the strains that showed elevated levels of persistence (Table 2), indicating a lower level of metabolic activity in these cells. These strains all have quality control present, but their stringent response has been disrupted. Bacterial antibiotic persister cells are able to evade killing by antibiotics because they have a low metabolic state and antibiotics mostly target actively growing cells. Also, the strains that resulted in very low levels of persistence had a higher intracellular concentration of ppGpp. The stringent response is activated by RelA in response to amino acid stress by synthesizing the alarmone ppGpp, so these cells were able to respond to the nutritional stress and did not enter into a persistent state. The highest levels of antibiotic persistence that were observed were in the strains that have quality control present and are in the Δ*relA* background. This would indicate that these persister cells are independent of the production of ppGpp. The editing-deficient mutant, *pheT* G318W, in the Δ*relA* background grown in medium B had elevated intracellular levels of AMP and GMP, indicative of ATP and GTP hydrolysis compared to when this strain was grown in medium A. These data are broadly inversely correlated with the changes in levels of persistence (Fig. 1), indicating that when quality control is absent, the ability to enter into persistence is significantly reduced.

**TABLE 2:** Intracellular nucleotide concentrations^*a*^

		ATP (μM)	ADP (μM)	AMP (μM)	GTP (μM)	GDP (μM)	GMP (μM)	Cyclic di-GMP (μM)	ppGpp (μM)
WT *pheS/pheT*	*relA*^+^-A	338	428	991	51	237	1,100	1.1	10
	*relA*^+^-B	266	315	665	43	238	908	1.8	6.1
	Δ*relA*-A	238	219	245	32	117	265	0.5	2.8
	Δ*relA*-B	185	155	218	24	88	247	1.0	2.6
*pheT* G318W	*relA*^+^-A	252	329	745	36	187	925	1.4	3.1
	*relA*^+^-B	274	344	797	40	238	908	1.2	7.1
	Δ*relA*-A	243	225	175	31	126	201	0.7	2.2
	Δ*relA*-B	245	302	421	32	172	513	0.8	3.8
*pheS* A294G	*relA*^+^-A	195	174	201	32	160	309	3.7	3.2
	*relA*^+^-B	161	162	253	22	143	419	1.9	2.0
	Δ*relA*-A	206	187	229	26	109	244	0.6	2.3
	Δ*relA*-B	133	136	179	16	85	177	0.7	1.9
*pheS* A294S	*relA*^+^-A	179	181	247	31	165	462	3.0	3.9
	*relA*^+^-B	152	188	279	26	158	455	7.0	3.5
	Δ*relA*-A	194	176	234	25	116	289	0.4	1.9
	Δ*relA*-B	101	104	141	13	89	159	1.2	2.7

a Both E. coli MG1655 *relA*^+^ and Δ*relA* strains were grown in medium A (containing all 20 amino acids) and medium B (containing no Phe, a lower Tyr concentration, and added *m*-Tyr) for all 4 PheRS strains. Concentrations are an average from biological triplicates and experimental triplicates.

The active site mutant, *pheS* A294S, in the Δ*relA* background grown in medium B had the highest level of antibiotic persistence and also had a consistently lower nucleotide pool than the other *pheS* A294S strains. It was also observed that when the stringent response is suppressed, which correlated with higher levels of persistence, there is a lower concentration of the secondary messenger cyclic di-GMP. These data support the idea that the strains that enter into antibiotic persistence have an overall lower intracellular metabolite pool.

### Proteome homeostasis is disrupted in strains that have a higher rate of antibiotic persistence.

**FIG 3:**
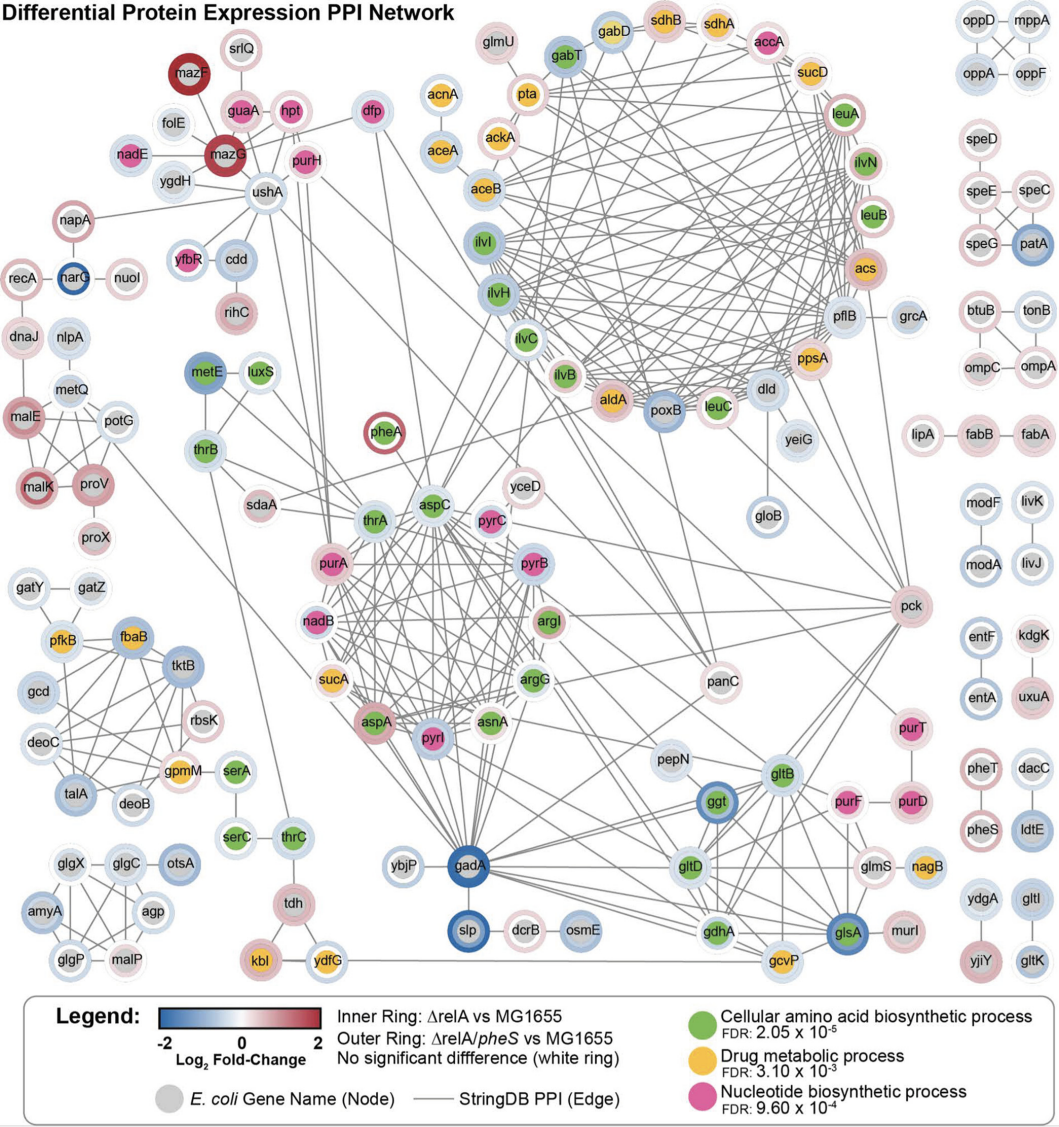
Protein homeostasis is disrupted during bacterial antibiotic persistence. Differential protein expression protein-protein interaction (PPI) network was performed for wild-type *pheS*/*pheT* and *pheS* A294S in *relA*^+^ and Δ*relA* backgrounds grown in medium B. The differentially expressed proteins are represented by their fold change for either Δ*relA* wild-type *pheS*/*pheT* versus *relA*^+^ wild-type *pheS*/*pheT* (inner ring) or Δ*relA pheS* A294S versus *relA*^+^ wild-type *pheS*/*pheT* (outer ring). The E. coli gene name is shown in the node, and cellular processes for amino acid biosynthesis (green), drug metabolism (yellow), and nucleotide biosynthesis (pink) are highlighted. Edges are derived from previously determined protein-protein interactions within StringDB.

Total proteome analyses were performed by using wild-type *pheS*/*pheT* and *pheS* A294S strains in both the *relA*^+^ and Δ*relA* backgrounds and grown in medium B to mid-log phase. After the proteins were digested, the smaller peptides were injected for LC-MS/MS analysis. There were a total of 2,351 proteins quantified across all samples including PheS, PheT, and PheA. These 3 proteins either had no change or were slightly overexpressed in the strains tested, which indicates that the expression of these proteins has not been affected by our strain construction or growth conditions. Overall, proteome homeostasis was disrupted for all strains compared to the wild-type *pheS*/*pheT* in the *relA*^+^ background (see Fig. S3A to E and Data Set S1 in the supplemental materials). Upon further investigation, we choose to focus the data analyses on the wild-type *pheS*/*pheT* in the *relA*^+^ and Δ*relA* background and *pheS* A294S in the Δ*relA* background. The differentially expressed proteins for wild-type *pheS*/*pheT* between *relA*^+^ and Δ*relA* (Table S1) and *pheS* A294S mutant in the Δ*relA* background versus the wild-type *pheS*/*pheT* in the *relA*^+^ background (Table S2) were used to develop a protein-protein interaction (PPI) network (Fig. 3).

10.1128/mBio.01132-21.4 FIG S3 Disruption of proteome homeostasis represented by volcano plots. Total proteome analysis was performed on wild-type *pheS* and *pheS* A294S in either MG1655 *relA*^+^ or Δ*relA* backgrounds grown in medium B. (A) *relA*^+^ *pheS* A294S versus *relA*^+^ wild-type *pheS*. (B) Δ*relA* wild-type *pheS* versus *relA*^+^ wild-type *pheS*. (C) Δ*relA pheS* A294S versus *relA*^+^ *pheS* A294S. (D) Δ*relA pheS* A294S versus Δ*relA* wild-type *pheS*. (E) Δ*relA pheS* A294S versus *relA*^+^ wild-type *pheS*. Download FIG S3, PDF file, 0.1 MB. Copyright © 2021 Wood et al. 2021 Wood et al. https://creativecommons.org/licenses/by/4.0/ This content is distributed under the terms of the Creative Commons Attribution 4.0 International license.

10.1128/mBio.01132-21.6 TABLE S1 Differentially expressed proteins in Δ*relA* WT *pheS/pheT* versus *relA*^+^ WT *pheS/pheT* grown in medium B. Download Table S1, DOCX file, 1.8 MB. Copyright © 2021 Wood et al. 2021 Wood et al. https://creativecommons.org/licenses/by/4.0/ This content is distributed under the terms of the Creative Commons Attribution 4.0 International license.

10.1128/mBio.01132-21.7 TABLE S2 Differentially expressed proteins in Δ*relA* *pheS* A294S versus *relA*^+^ WT *pheS*/*pheT* grown in medium B. Download Table S2, DOCX file, 1.8 MB. Copyright © 2021 Wood et al. 2021 Wood et al. https://creativecommons.org/licenses/by/4.0/ This content is distributed under the terms of the Creative Commons Attribution 4.0 International license.

10.1128/mBio.01132-21.8 DATA SET S1 Total proteome analysis. Changes in protein abundance were monitored for wild-type *pheS* and *pheS* A294S in either MG1655 *relA*^+^ and Δ*relA* backgrounds grown in medium B. Over- and underrepresented proteins are noted. Download Data Set S1, XLSX file, 1.2 MB. Copyright © 2021 Wood et al. 2021 Wood et al. https://creativecommons.org/licenses/by/4.0/ This content is distributed under the terms of the Creative Commons Attribution 4.0 International license.

The PPI network showed several proteins that were differentially expressed in pathways for cellular amino acid biosynthesis, drug metabolism, and nucleotide biosynthesis (represented by the green, yellow, and pink nodes, respectively, in Fig. 3). In general, the proteins involved in amino acid biosynthesis were downregulated in the *pheS* A294S strains (represented by the outer ring in Fig. 3) and were either slightly downregulated or had no change in the wild-type *pheS*/*pheT* (represented by the inner ring in Fig. 3). These results are consistent with *pheS* A294S having a higher fraction of persisters than the wild-type *pheS*/*pheT* in the Δ*relA* background compared to the *relA*^+^ background (Fig. 1). Strikingly, MazF and MazG were both enriched in all strains compared to the wild-type *pheS/pheT* in the *relA*^+^ background. MazF is an RNase toxin that comprises part of the MazEF toxin-antitoxin module which has been previously studied in persister formation in E. coli (33, 34). MazG is a broad-specificity nucleoside triphosphatase (NTPase) that modulates MazEF and is involved in regulating cell survival under amino acid starvation conditions (35, 36).

## DISCUSSION

### Aminoacyl-tRNA synthetase quality control is a determinant for antibiotic persistence.

Aminoacyl-tRNA synthetase quality control is important for accurate synthesis of the proteome. When quality control is absent, misacylation of tRNAs can occur, which was once thought to always be detrimental to the cell, but it is now understood that in certain cases a low level of mistranslation can be beneficial for the cell to adapt to a new or challenging environment (27, 37). It was previously observed that when PheRS quality control is abolished by the use of a PheRS editing-deficient E. coli strain, *pheT* G318W, the cells were not able to activate the stringent response (25). The stringent response is critical for bacteria to withstand amino acid stress, and it is activated when deacylated-tRNA enters the A-site of the ribosome which signals for RelA to begin synthesizing the alarmone (p)ppGpp (38). When the PheRS editing-deficient strain is grown under starvation conditions and in the presence of *m*-Tyr, a nonproteogenic amino acid that is produced from the oxidation of Phe, misacylated *m*-Tyr-tRNA^Phe^ falsely inhibits the stringent response from being triggered because it senses starvation via deacylated-tRNA and not misacylated-tRNA (25). The stringent response has been thought to be a determinant for antibiotic persistence; however, there have been a few studies where persistence has been shown to be stringent response independent (12, 13, 20).

In this study, we have investigated the effects of PheRS quality control on bacterial antibiotic persistence in E. coli. Along with the editing-deficient PheRS, we chose to study two different active site PheRS mutants, αA294G and αA294S. It is assumed that the αA294G mutation would have decreased substrate discrimination and the αA294S mutation would have increased discrimination (32). The αA294G PheRS is unable to discriminate between Phe and *m*-Tyr and has a 12.7-fold reduction in discrimination for Tyr compared to wild-type PheRS. However, αA294S PheRS has a 16.8-fold increase and a 2.6-fold increase of discrimination for *m*-Tyr and Tyr, respectively, compared to wild-type PheRS (Table 1). With these kinetic data, it can be hypothesized that αA294G PheRS will be able to efficiently synthesize *m*-Tyr-tRNA^Phe^; however, because editing is intact, the misacylated *m*-Tyr-tRNA^Phe^ will be moved into the editing site of PheRS where it will be hydrolyzed (see Fig. S4A in the supplemental material). αA294G PheRS also has decreased efficiency for phenylalanine adenylation, and so, in addition to quality control, this mutant also fails to efficiently synthesize Phe-tRNA^Phe^, and this would also contribute to increased levels of deacylated tRNA^Phe^. With the increased discrimination of αA294S PheRS, it will not be able to synthesize *m*-Tyr-tRNA^Phe^ (Fig. S4B). In both of these scenarios, the amount of the intracellular deacylated tRNA^Phe^ would increase, and under starvation conditions, it would activate the stringent response.

10.1128/mBio.01132-21.5 FIG S4 Molecular model of increased deacylated tRNA^Phe^ in the cell. (A) Reaction of active site mutant αA294G PheRS, which has decreased specificity for *m*-Tyr and Tyr compared to wild-type PheRS. The noncognate amino acid will be efficiently aminoacylated onto tRNA^Phe^, which will be moved to the editing site where hydrolysis of the misacylated tRNA^Phe^ will occur. Deacylated tRNA^Phe^ and noncognate amino acid will be released from PheRS. (B) Reaction of active site mutant αA294S PheRS, which has increased specificity for *m*-Tyr and Tyr compared to wild-type PheRS. The noncognate amino acid will not bind and therefore will not be aminoacylated onto tRNA^Phe^. Download FIG S4, PDF file, 0.1 MB. Copyright © 2021 Wood et al. 2021 Wood et al. https://creativecommons.org/licenses/by/4.0/ This content is distributed under the terms of the Creative Commons Attribution 4.0 International license.

Three different PheRS-encoding mutations, *pheT* G318W, *pheS* A294G, and *pheS* A294S, were made on the chromosome of E. coli MG1655 and the corresponding Δ*relA* strain. Intriguingly, there was a reduction in persistence in the *pheT* G318W Δ*relA* strain grown under amino acid limitation (Fig. 1 and Fig. S1). In this strain, the misacylated *m*-Tyr-tRNA^Phe^ is not edited and so the stringent response is not triggered since the amount of intracellular deacylated tRNA^Phe^ does not change. This correlates with our previous finding that when *pheT* G318W is unable to edit *m*-Tyr-tRNA^Phe^ this resulted in the stringent response being unable to be activated (25). These results seem to indicate that when the intracellular concentration of deacylated tRNA^Phe^ increases and a stringent response is able to be mounted, the cell responds appropriately; however, when RelA is not present, and the stringent response is disrupted, the cell can directly enter into a persistent state.

### Deacylated tRNA triggers antibiotic persistence independent of the stringent response.

Taken together, the kinetic data and the antibiotic persistence data suggest that the intracellular level of deacylated tRNA may ultimately be a trigger for persistence. To investigate this further, Northern blot analysis was performed on all 4 PheRS strains in both the *relA*^+^ and Δ*relA* E. coli background, which did confirm that the amount of aminoacylated tRNA^Phe^ significantly decreased in both *pheS* A294G and *pheS* A294S in the Δ*relA* background when grown in medium B (Fig. 2). This decrease in aminoacylated tRNA^Phe^ correlates with these two strains displaying the greatest persistence. Our data support that the increase in persistence in the Δ*relA* E. coli background is consistent with an increase in intracellular deacylated tRNA^Phe^ concentrations and is independent of the stringent response. We attempted to further investigate persistence and the connection to deacylated tRNA accumulation using a *pheS* A294G/*pheT* G318W double mutant, but this strain had a severe growth defect when grown in medium B, and this growth defect was even more pronounced in the Δ*relA* background (Fig. S2).

Other studies have proposed that when aaRS activity is restricted or inhibited, the amount of deacylated tRNA in the cell would increase and this may be the trigger for antibiotic persistence, consistent with our data shown here (16, 20–22). In several previous studies, it was shown that GluRS was inactivated by phosphorylation via the toxin HipA, leading to limited tRNA^Glu^ aminoacylation and increased levels of persistence. When this occurs, the amount of deacylated tRNA^Glu^ increases and it enters the A-site of the ribosome, triggering the stringent response via RelA; thus, this mechanism of persistence is RelA dependent (16, 22). Interestingly, a few of these studies were conducted in bacteria that lack RelA and have a disrupted stringent response. One study was conducted using Chlamydia, a Trp auxotroph, and the authors used indolmycin to inhibit TrpRS, which led to a decrease in aminoacylated tRNA^Trp^ that reduced translation rates and increased persistence. Since Chlamydia does not encode RelA or SpoT, this mechanism of persistence is stringent response independent (20). A few studies have been conducted in Caulobacter crescentus, which relies solely on SpoT to regulate the stringent response which requires both amino acid limitation and carbon or nitrogen starvation. In one study, HipA was able to phosphorylate both GluRS and TrpRS, which inhibited their aminoacylation activity, and upon a further carbon or nitrogen stress, SpoT would synthesize (p)ppGpp and the cells would enter into a persister state (21). In another study, the HipA phosphorylation of TrpRS was further investigated, and it was found that as the levels of Trp increase in the cell because it is not being used to aminoacylate tRNA^Trp^, this led to the inhibition of GlnE, which reduced the amount of glutamine production, creating a nitrogen shortage. This imbalance in amino acids ultimately resulted in an increase in persister cells that is SpoT dependent (39).

There has also been a recent study showing that defects in tRNA, such as a lack in methylation, seem to have a link to antibiotic resistance and persistence (40, 41). In the present study, the highest levels of persistence were associated with increased levels of deacylated tRNA^Phe^ and when RelA was deleted. SpoT is encoded in our Δ*relA* strains, accounting for our ability to detect ppGpp. In all the Δ*relA* strains, ppGpp levels remained steady regardless of amino acid limitation, suggesting that SpoT activity was not significantly altered when persistence was increased.

### Changes in metabolism reveal a cellular reprogramming in antibiotic persister cells.

Metabolomics and proteomics were performed in order to further understand the metabolic state of the different strains that were used in this study. The metabolomics data showed an overall trend of decreased intracellular concentrations of nucleotides compared to the wild-type strain (Table 2). For example, the strain with the highest level of persistence, Δ*relA pheS* A294S grown in medium B, had a reduced concentration of the nucleotides tested except for cyclic di-GMP and ppGpp compared to the other *pheS* A294S strains. This is consistent with antibiotic persister cells having a low biochemical and metabolic state because they are in a dormant, nongrowing form. There was also a general trend of AMP and GMP having a higher intracellular concentration compared to their di- and triphosphate purine nucleotides, which is also consistent with persistent cells being in a low energetic state.

The proteomics data showed that cellular homeostasis was disrupted for wild-type *pheS*/*pheT* and *pheS* A294S in the Δ*relA* background compared to wild-type *pheS*/*pheT* in the *relA*^+^ background when grown in medium B (Fig. 3). From the PPI network, it was observed that most of the proteins that are involved in amino acid biosynthesis were underrepresented compared to wild-type *pheS*/*pheT* in the *relA*^+^ background which is consistent with the cells entering into a dormant state. Furthermore, from the proteomics data PheA was overexpressed in *pheS* A294S in the Δ*relA* background. Expression of PheA is required for the biosynthesis of phenylalanine and is regulated by transcription attenuation by the synthesis of the leader peptide PheL. Under normal conditions, PheRS maintains the level of Phe-tRNA^Phe^ for the attenuation of *pheA* transcription (25, 42). However, *pheS* A294S in the Δ*relA* background led to an increased amount of deacylated tRNA^Phe^ when grown in medium B, and this also led to the increased expression of PheA, which further supports our model. Furthermore, PheA had normal levels of expression when comparing the proteomics data of wild-type *pheS*/*pheT* in the Δ*relA* background compared to the *relA*^+^ background, indicating that deletion of *relA* does not affect the transcription of *pheA*.

The toxin MazF was significantly enriched in both wild-type *pheS*/*pheT* and *pheS* A294S in the Δ*relA* background, and the antitoxin MazE was not detected above the limit of detection. MazEF is a type II toxin-antitoxin module in which when the labile antitoxin, MazE, is degraded in the cell MazF can cause toxicity by cleaving mRNA, which may occur independent of translation (43). The regulator of the MazEF module, MazG, was also upregulated. MazG is an NTP hydrolase that can cleave (p)ppGpp produced by RelA or SpoT and also hydrolyze the NTP substrates for the synthesis of (p)ppGpp (35, 36). Since the upregulated MazG was observed in the Δ*relA* background, it could be hypothesized that the (p)ppGpp that MazG would be degrading is being produced from SpoT. It is also possible that MazG might be degrading NTPs to prevent the synthesis of (p)ppGpp, since it was observed from the metabolomics that ATP and GTP concentrations were reduced in these strains. Furthermore, to ensure that the increase in the expression levels of MazF and MazG was not an artifact of transcriptional read-through from the disruption of *relA* using a kanamycin cassette, sequencing of this operon was performed (data not shown). We confirmed that both the 5′ untranslated region (UTR) and 3′ end of *relA* are intact and that the 5′ region of *mazEF*, which contains both of its promoters, was not disrupted by the kanamycin insertion into the *relA* gene. Both of these promoters are required for the autoregulation of *mazEF*, and when MazE is degraded, MazF is released as a toxin (44, 45). The promoter for *mazG* was also not disrupted, which indicates that the increased levels of expression for both MazF and MazG are due to the cells having entered into an antibiotic persister state. There were also changes in the levels of production for numerous proteins involved in fatty acid synthesis, purine biosynthesis, and the tricarboxylic acid cycle, which have previously been shown to be disrupted when bacterial persistence is triggered (46–48). It should also be noted that for both the metabolomics and proteomics experiments, the entire bacterial population was used for the metabolite and protein extractions, so the quantifications are an average of the population. Since bacterial persisters represent only a small fraction of the entire population (∼1 to 10% in this study), these subtle changes in the metabolite concentration and protein expression may be more pronounced in the persister cell. Naturally occurring or spontaneous persister cells occur at a frequency of ∼1 × 10^−6^, and mistranslation occurs every ∼1 × 10^−4^ codons (27, 49). As these small frequencies in error begin to accumulate, so the rate of bacterial persister formation increases.

Our data reveal a mechanism for persister formation where reduced translation capacity is accompanied by accelerated depletion of cellular NTP pools, which in turn triggers antibiotic persistence. Our data further show that the frequency of persister formation via this mechanism is significantly influenced by the specificity and efficiency with which substrates for translation are synthesized. While in our study changes in PheRS specificity and efficiency were achieved via genetic manipulation, nutrient depletion can also have comparable effects on aminoacyl-tRNA synthesis in wild-type cells (50). This mechanism of bacterial persister formation is ppGpp independent. Our study and several other studies have recently been challenging the role of RelA and ppGpp in persister formation. Instead, changes in amino acid concentrations, inhibition of aaRSs, and slowed translation are all mechanisms now known to be able to induce bacterial antibiotic persistence. Taken together, these and previous findings suggest that small amino acid imbalances, while not significantly impacting the population as a whole, could lead to heterogenous quality control outcomes that affect a small number of cells, thereby triggering the formation of a subpopulation of antibiotic persisters.

## MATERIALS AND METHODS

### Strains, plasmids, and general methods.

All strains were constructed in either a *relA*^+^ E. coli (MG1655) background or a Δ*relA* E. coli background (51). An editing-deficient PheRS E. coli strain, *pheT* G318W, was previously made (25, 29). Two different mutant E. coli strains, *pheS* A294G and *pheS* A294S, were constructed using scarless Cas9-assisted recombineering (no-SCAR) (30, 31). Briefly, pKDsgRNA-*pheS* was made using round-the-horn cloning with pKDsgRNA-*ackA*, and primers for PCR were as follows: F-sgRNA pheS, 5′-ATCGACCCGGAAGTTTACTCGTTTTAGAGCTAGAAATAGCAAGTTAAAATAAGG-3′, and PtetR, 5′-PO_4_-GTGCTCAGTATCTCTATCACTGA-3′. After pCas9Cr4 was chemically transformed into the host strain, pKDsgRNA-*pheS* was transformed by electroporation. Colonies that contained both plasmids were grown in super optimal broth medium with shaking at 30°C. When the optical density at 600 nm (OD_600_) reached 0.1 to 0.2, λ-red expression was induced with 0.2% arabinose. Once the culture reached an OD_600_ of 0.4 to 0.6, 2 μM single-stranded DNA (ssDNA) recombineering oligonucleotide that contained the desired mutation was transformed by electroporation. The *pheS* A294G oligonucleotide sequence was 5′-AACGCAACATAGTCAGACGCTCCATCCCCATCCCAAACCCAAAACCGCTATAAACTTCGGGATCAATGCCAACGTTACGC-3′, and the *pheS* A294S oligonucleotide sequence was 5′-AACGCAACATAGTCAGACGCTCCATCCCCATCCCAAAGCTAAAACCGCTATAAACTTCGGGATCAATGCCAACGTTACGC-3′. Both recombineering oligonucleotides had four phosphorothioate bonds at the 5′ end of the oligonucleotide. Recombineering occurred at 30°C for 2 h while shaking. Counterselection is achieved by induction of Cas9 with anhydrotetracycline. pKDsgRNA-*pheS* and pCas9Cr4 curing was performed exactly as described previously (31). Clones were screened by colony PCR using forward primer 5′-CTCGCAGAACTGGTTGCCAG-3′ and reverse primer 5′-CACGCAGTTTGTCAGCGTTCG-3′. Clones were confirmed by DNA sequencing using forward primer 5′-CTGATTGTTGATACCAACATC-3′. E. coli XL1-Blue/pQE31-FRS (producing His_6_-tagged wild-type PheRS) and E. coli XL1-Blue/pQE31-*pheS*A294G (producing His_6_-tagged αA294G PheRS) were previously constructed (52). A point mutation was made in the *pheS* gene of pQE31-FRS by PCR-based site-directed mutagenesis using two self-complementary primers (5′-ACTCTGGTTTCAGCTTCGGGATGG-3′) to generate E. coli XL1-Blue/pQE31-*pheS*A294S (producing His_6_-tagged αA294S PheRS). The mutation was confirmed by DNA sequencing. Protein expression and purification were performed essentially as described previously (53).

### ATP/PP_i_ exchange.

Detailed description of the methods for ATP/PP_i_ exchange to determine steady-state kinetics of amino acid activation are in Text S1 in the supplemental material.

10.1128/mBio.01132-21.1 TEXT S1 Methods and Materials. Download Text S1, DOCX file, 0.02 MB. Copyright © 2021 Wood et al. 2021 Wood et al. https://creativecommons.org/licenses/by/4.0/ This content is distributed under the terms of the Creative Commons Attribution 4.0 International license.

### Growth medium.

Lysogeny broth (LB) was made with 0.5% NaCl, 0.5% yeast extract, and 1% tryptone. A supplemented M9 minimal medium that was used for persister assays was developed based on a previous protocol (14). A 2× supplemented M9 minimal medium was prepared as follows: 2× M9 salts, 0.8% glucose, 4 mM MgSO_4_, 0.2 mM CaCl_2_, 2 μg/ml thiamine, 1 μg/ml FeSO_4_ solution, 80 μg/ml all proteogenic amino acids except for phenylalanine and tyrosine. For the persister assays, two different media were used, medium A and medium B. Medium A contained 1× supplemented M9 minimal medium, 40 μg/ml phenylalanine, and 40 μg/ml tyrosine. Medium B contained 1× supplemented M9 minimal medium, 10 μg/ml tyrosine, and 40 μg/ml *m*-tyrosine (no phenylalanine was used in this medium).

### Minimum duration of killing (MDK) persister assays.

MDK persister assays were performed as described in several studies (3, 4, 14, 54). Overnight cultures were grown in LB and were then diluted 1:100 into 6 ml of either medium A or medium B in culture tubes. The cultures were grown at 37°C while shaking at 250 rpm until they reached early exponential phase (OD_600_ = 0.2 to 0.3). At this point 1 ml of culture was taken out, serially diluted, and plated on LB plates to count CFU; 100 μg/ml ampicillin was added to the remaining culture; and growth continued at 37°C with shaking. Every hour for 3 h, 1 ml of culture was removed, washed in sterile phosphate-buffered saline (PBS) twice to remove the antibiotic, serially diluted, and plated on LB plates. To calculate persisters, the CFU of survivors after ampicillin treatment was divided by the CFU of the culture before ampicillin treatment.

### Quantification of aminoacylated and deacylated tRNA.

Purification of total tRNA, acid/urea gel electrophoresis, and Northern blotting methods are described in detail in Text S1.

### Targeted metabolomic analysis and quantification.

Metabolites were extracted from exponentially growing E. coli cells as described previously (55, 56). Detailed methods for the metabolite extraction are described in Text S1.

LC-MS analysis of the metabolites was performed as described previously (57). The dried metabolites were suspended in 50 μl of MilliQ water and centrifuged at 16,000 × *g* for 30 min at 4°C, and the supernatant was transferred to an LC vial. Separation of metabolites was performed on a Thermo Scientific UltiMate 3000 ultra-high performance liquid chromatography system equipped with a zwitter-ionic-phosphorylcholine hydrophilic interaction liquid chromatography, 150- by 2.1-mm, 3-μm column with a flow rate of 0.15 ml/min and a column temperature of 37°C; mobile phase A was 10 mM ammonium acetate, pH 6, and mobile phase B was 10% 10 mM ammonium acetate, pH 6, 90% acetonitrile. Chromatography gradient was as follows: isocratic 100% mobile phase B for 3 min, linear gradient to 20% B for 22 min, linear gradient to 100% B for 1 min, isocratic 100% B for 1 min, linear gradient to 20% B for 8 min, linear gradient to 100% B for 5 min, and isocratic 100% B for 15 min. Standard curves with a mixture of ATP, ADP, AMP, GTP, GDP, GMP, cyclic di-AMP, cyclic di-GMP, and ppGpp ranging from 0 to 5 ppm spiked with 10 ppm internal standard mix were done before and after each experimental set to ensure column integrity. The MS was performed on a Thermo Scientific TSQ Quantiva (triple-stage quadrupole MS) with a spray voltage of 3,500 V in the negative ion mode, ion vaporizer at 50°C, and ion transfer tube at 350°C. Analysis and quantification were performed using Xcalibur data acquisition and interpretation software. Intracellular concentrations were calculated assuming an OD_600_ of 1.0 equals 8 × 10^8^ cells and that the volume of one E. coli cell growing in exponential phase equals 1 × 10^−15^ liter (58, 59). This was then normalized by the extraction efficiency for [^13^C]ATP and [^13^C]GTP.

### Protein digestion and mass spectrometry. (i) Digestion of intact E. coli for shotgun proteomics.

Twenty-milliliter cultures were inoculated to a starting OD at 600 nm of 0.01 in either medium A or medium B using an overnight culture to stationary phase. After reaching mid-log, cells were chilled on ice and pelleted by centrifugation for 2 min at 8,000 rpm. The resulting pellet was frozen at −80°C for downstream processing. For cell lysis and protein digest, cell pellets were thawed on ice and 2 μl of cell pellet was transferred to a microcentrifuge tube containing 40 μl of lysis buffer (10 mM Tris-HCl, pH 8.6, 10 mM dithiothreitol [DTT], 1 mM EDTA, and 0.5% antilymphocyte serum [ALS]). Cells were lysed by vortex for 30 s, and disulfide bonds were reduced by incubating the reaction mixture for 30 min at 55°C. The reaction was briefly quenched on ice, and 16 μl of a 60 mM iodoacetamide solution was added. Alkylation of cysteines proceeded for 30 min in the dark. Excess iodoacetamide was quenched with 14 μl of a 25 mM DTT solution, and the sample was then diluted with 330 μl of 183 mM Tris-HCl buffer (pH 8.0) supplemented with 2 mM CaCl_2_. Proteins were digested overnight using 12 μg sequencing-grade trypsin. Following digestion, the reaction was then quenched with 12.5 μl of a 20% trifluoroacetic acid (TFA) solution, resulting in a sample pH of <3. Remaining ALS reagent was cleaved for 15 min at room temperature. The sample (∼30 μg protein) was desalted by reverse-phase cleanup using C_18_ UltraMicroSpin columns. The desalted peptides were dried at room temperature in a rotary vacuum centrifuge and reconstituted in 20 μl 70% formic acid-0.1% TFA (3:8 [vol/vol]) for peptide quantitation by UV_280_. The sample was diluted to a final concentration of 0.4 μg/μl, and 5 μl (2 μg) was injected for LC-MS/MS analysis.

### (ii) Data acquisition and analysis.

LC-MS/MS was performed using an Acquity UPLC M-class (Waters) and Q Exactive Plus mass spectrometer. The analytical column employed was a 65-cm-long, 75-μm-internal-diameter PicoFrit column (New Objective) packed in-house to a length of 50 cm with 1.9-μm ReproSil-Pur 120-Å C_18_-AQ (Dr. Maisch) using methanol as the packing solvent. Peptide separation was achieved using mixtures of 0.1% formic acid in water (solvent A) and 0.1% formic acid in acetonitrile (solvent B) with a 90-min gradient of either 0/1, 2/7, 60/24, 65/48, 70/80, 75/80, 80/1, or 90/1 (min/% B, linear ramping between steps). Gradient was performed with a flow rate of 250 nl/min. At least one blank injection (5 μl 2% B) was performed between samples to eliminate peptide carryover on the analytical column. One hundred femtomoles of trypsin-digested bovine serum albumin (BSA) or 100 ng trypsin-digested wild-type K-12 MG1655 E. coli proteins was run periodically between samples as quality control standards.The mass spectrometer was operated with the following parameters: (MS1) 70,000 resolution, 3e^6^ automatic gain control (AGC) target, 300 to 1,700 *m/z* scan range; (data-dependent MS2) 17,500 resolution, 1e^6^ AGC target, top 10 mode, 1.6 *m/z* isolation window, 27 normalized collision energy, 90-s dynamic exclusion, unassigned, and +1 charge exclusion. Data were searched using MaxQuant version 1.6.10.43 with deamidation (NQ), oxidation (M), and phospho (STY) as variable modifications and carbamidomethyl (C) as a fixed modification with up to 3 missed cleavages, 5-amino-acid (aa) minimum length, and 1% false-discovery rate (FDR) against a UniProt E. coli database. Search results were analyzed with Perseus version 1.6.2.2.
