# Supplementary figures and images for: Deacylated tRNA Accumulation Is a Trigger for Bacterial Antibiotic Persistence Independent of the Stringent Response
Source: mBio. 2021 Jun 15;12(3):e01132-21. doi: 10.1128/mBio.01132-21 (PMC8262941; doi:10.1128/mBio.01132-21)

A.

**WT *pheS/pheT***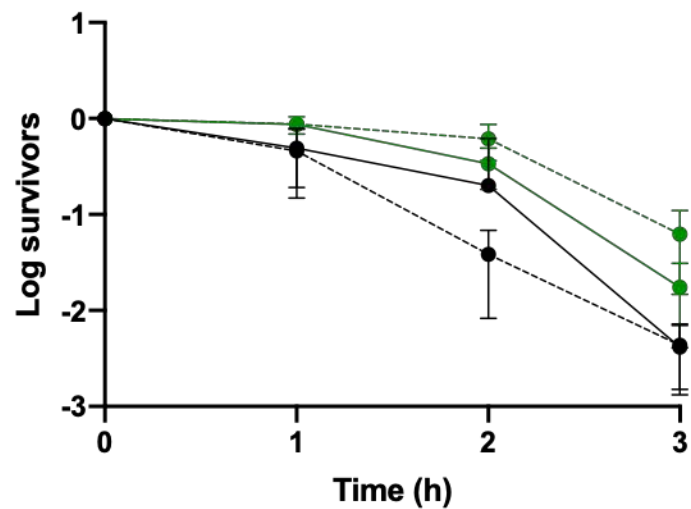

B.

***pheT* G318W**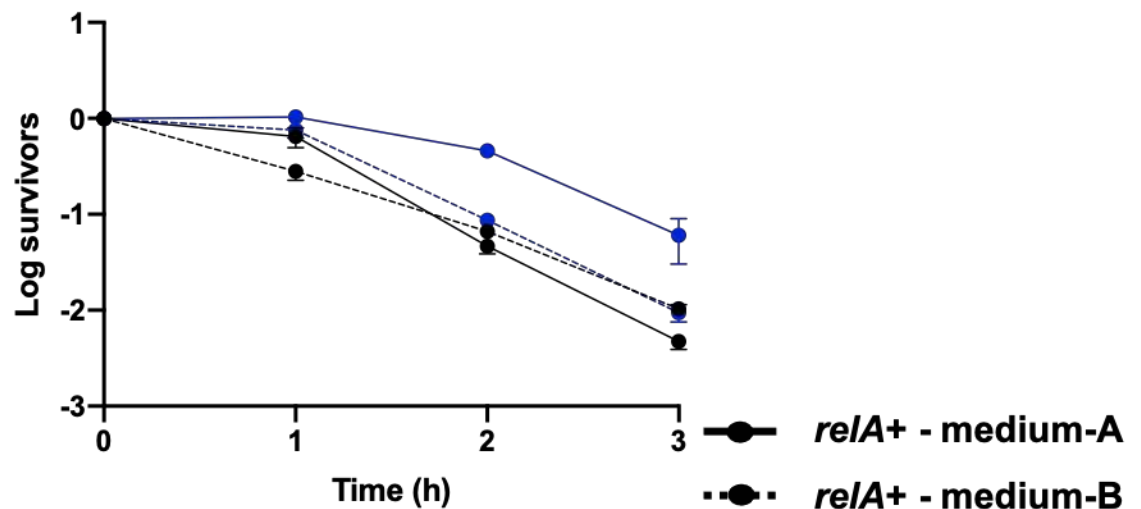

C.

***pheS* A294G**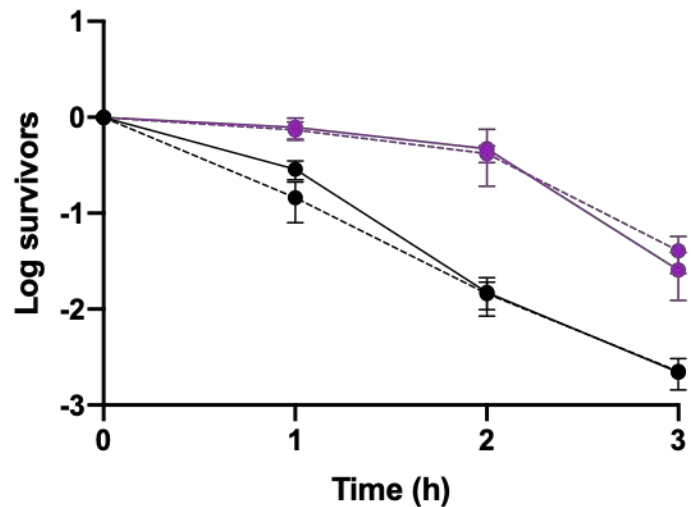

D.

***pheS* A294S**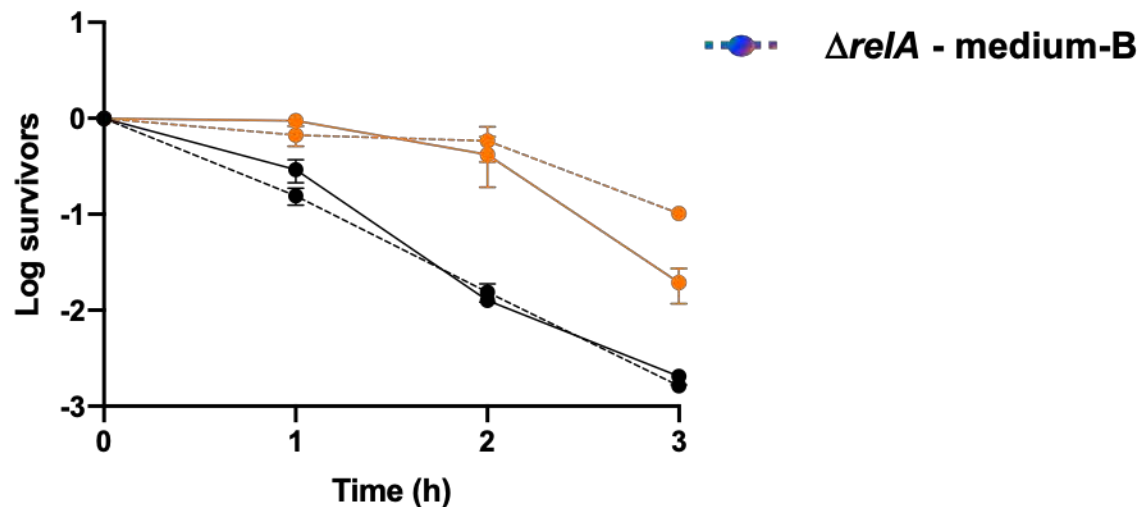

Supplement: FIG S1 [file mbio.01132-21-sf001.pdf]

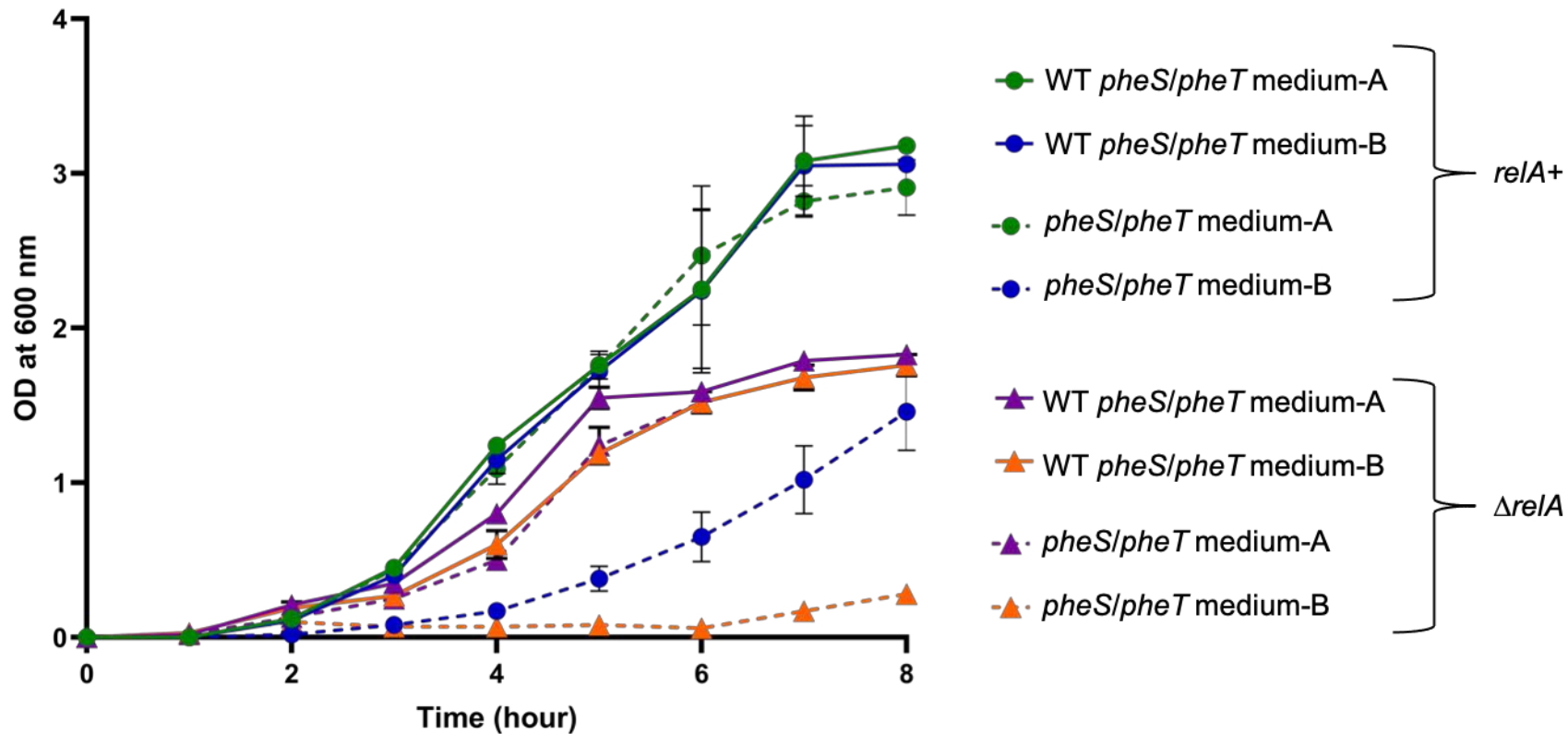

Supplement: FIG S2 [file mbio.01132-21-sf002.pdf]

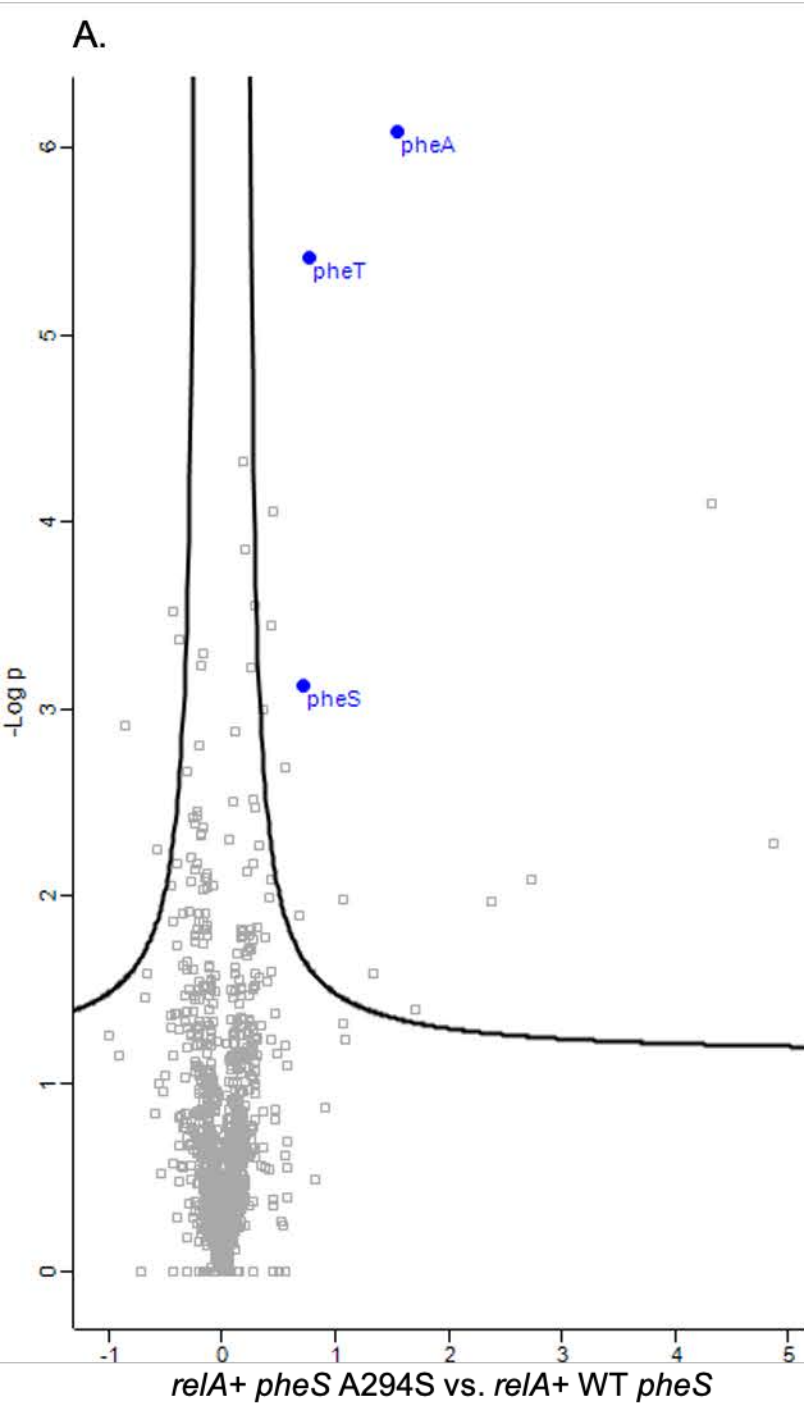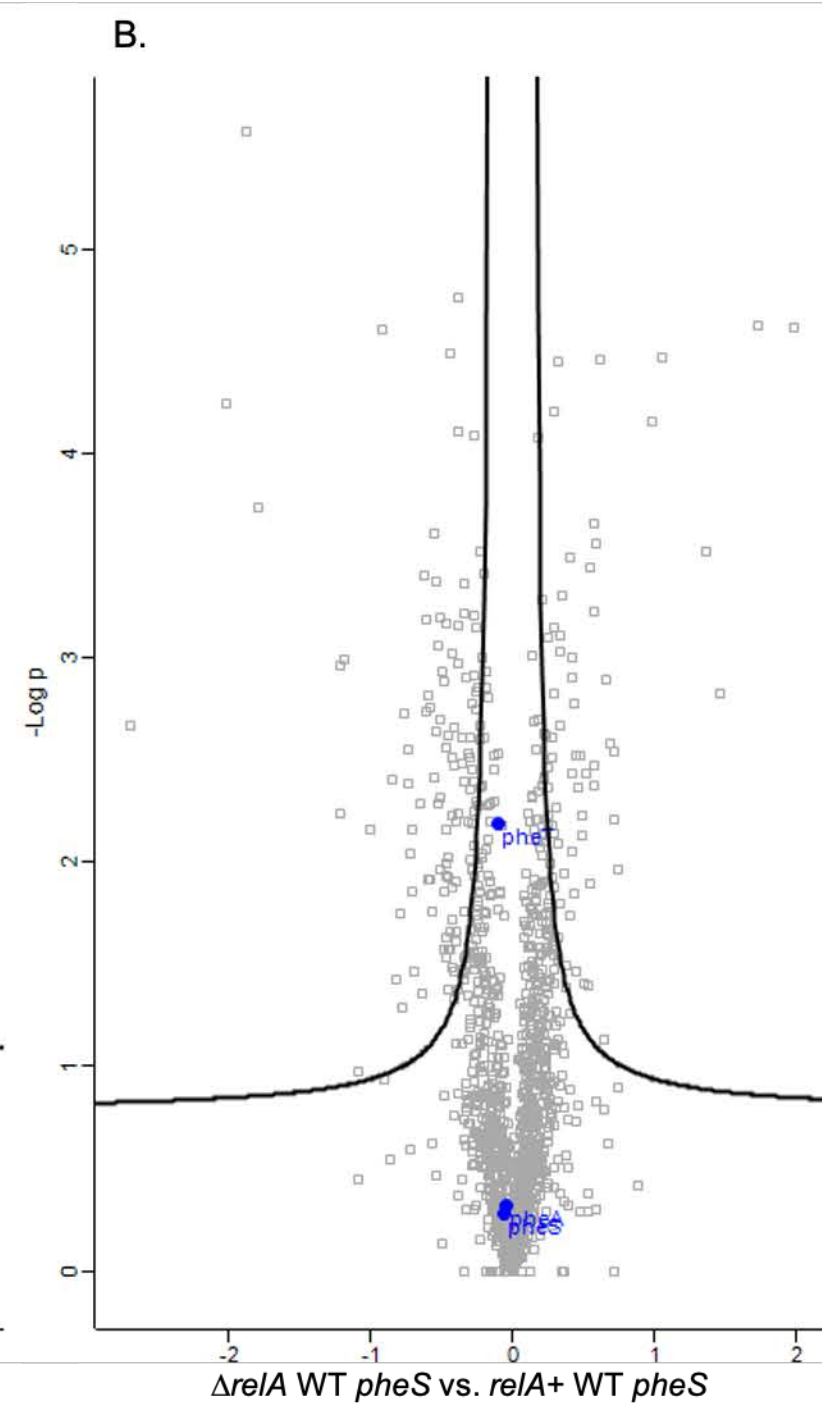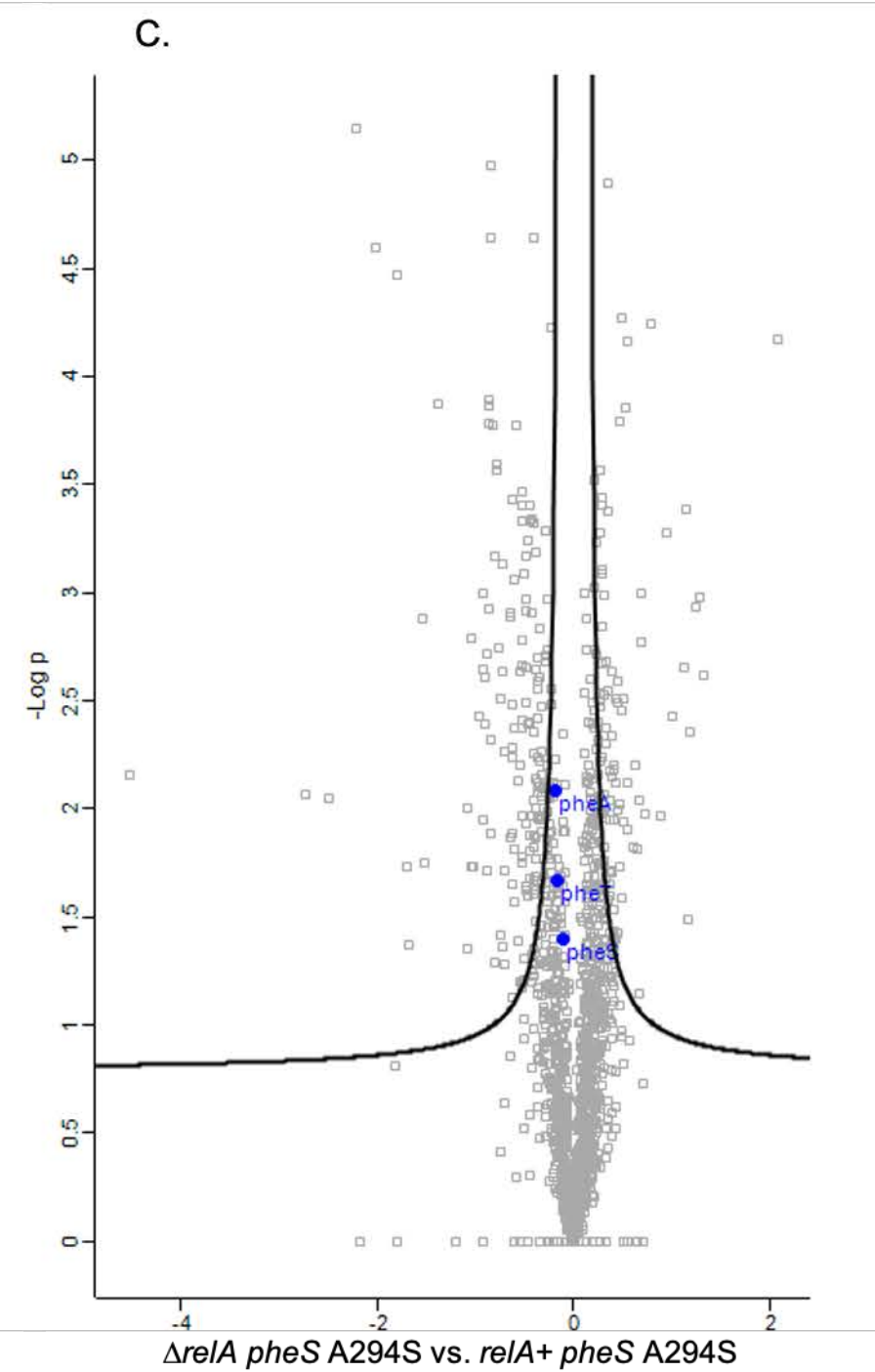

D.

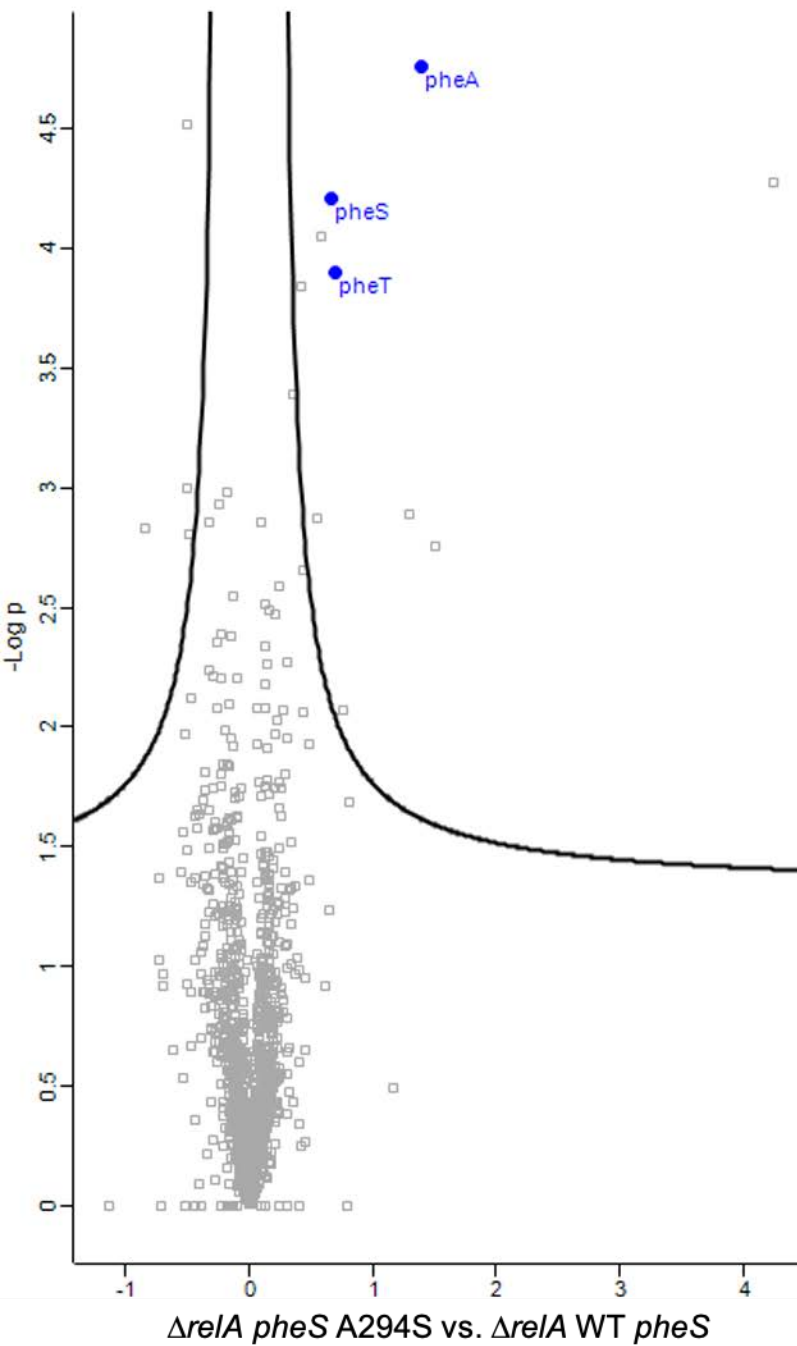

E.

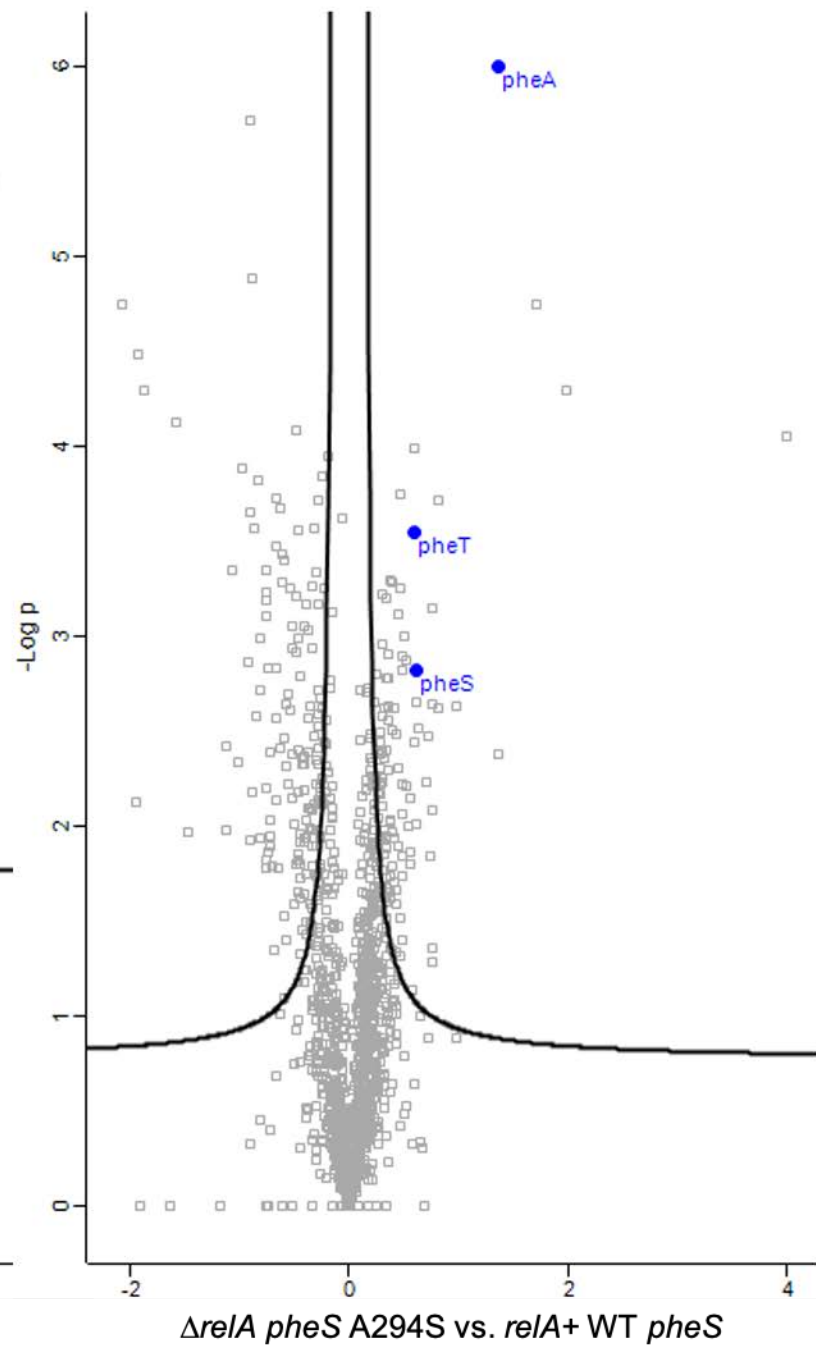

Supplement: FIG S3 [file mbio.01132-21-sf003.pdf]
