# Supplementary material for: Deacylated tRNA Accumulation Is a Trigger for Bacterial Antibiotic Persistence Independent of the Stringent Response
Source: mBio. 2021 Jun 15;12(3):e01132-21. doi: 10.1128/mBio.01132-21 (PMC8262941; doi:10.1128/mBio.01132-21)
Supplement: TABLE S1 [file mbio.01132-21-st001.docx]

**Table S1. Differentially expressed proteins in *ΔrelA* WT *pheS/pheT* vs. *relA*+ WT *pheS/pheT* grown in medium-B.**

**
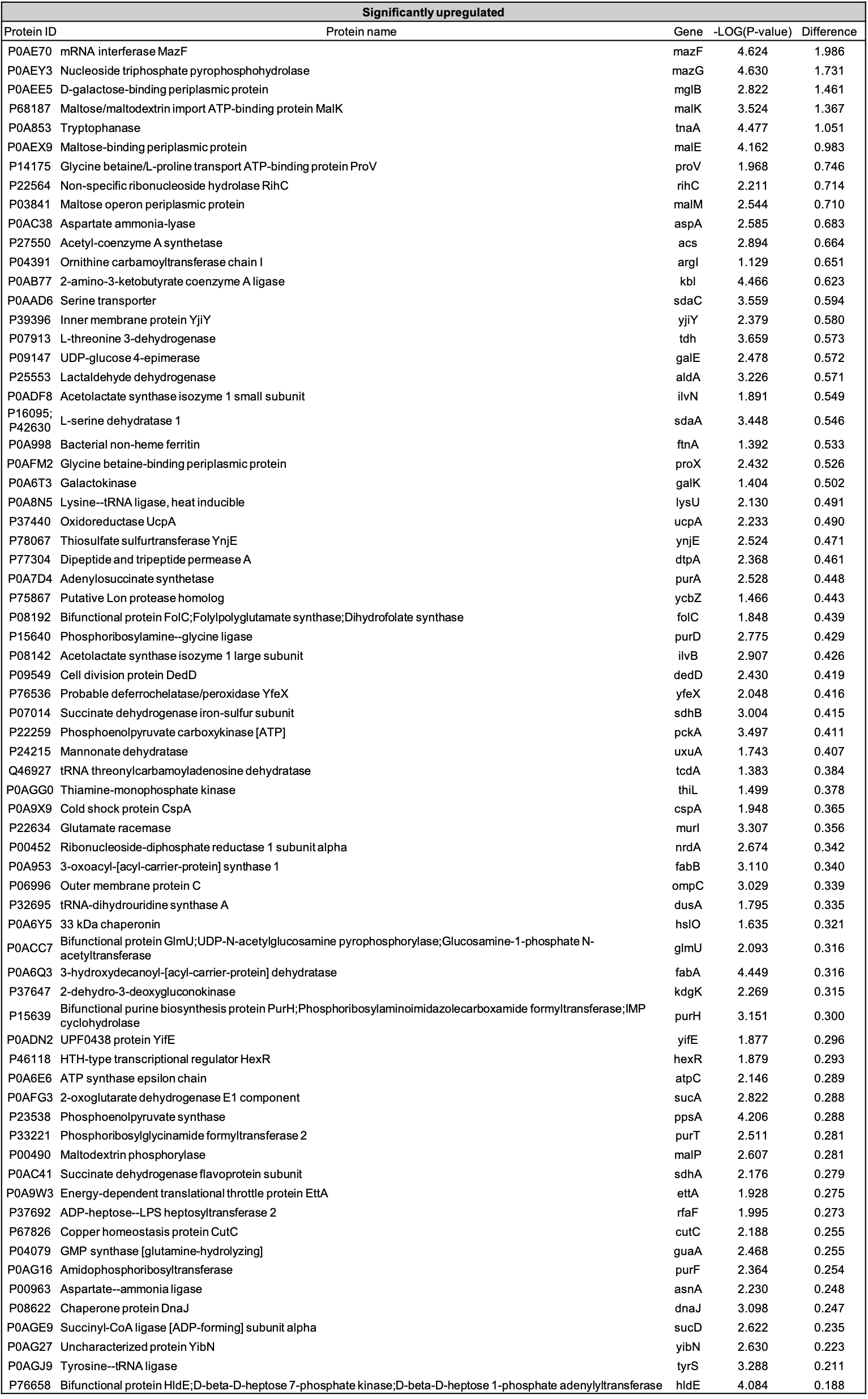
**

**
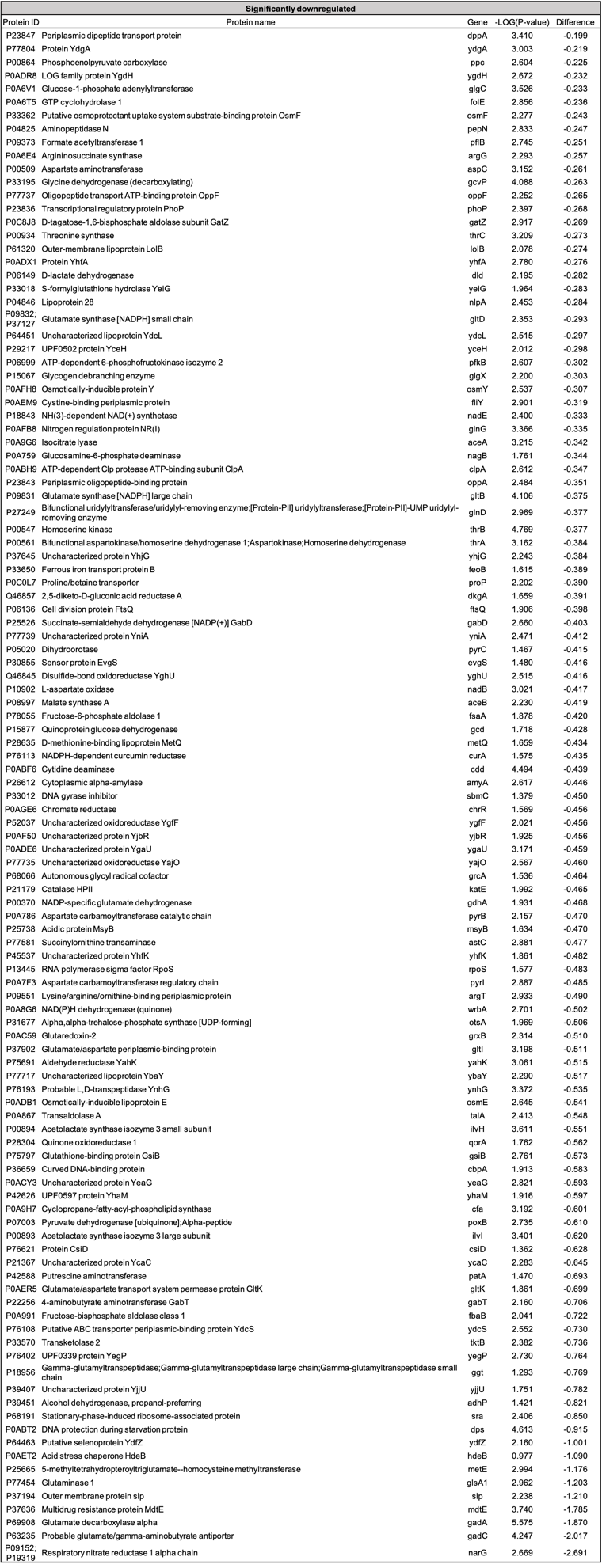
**

**
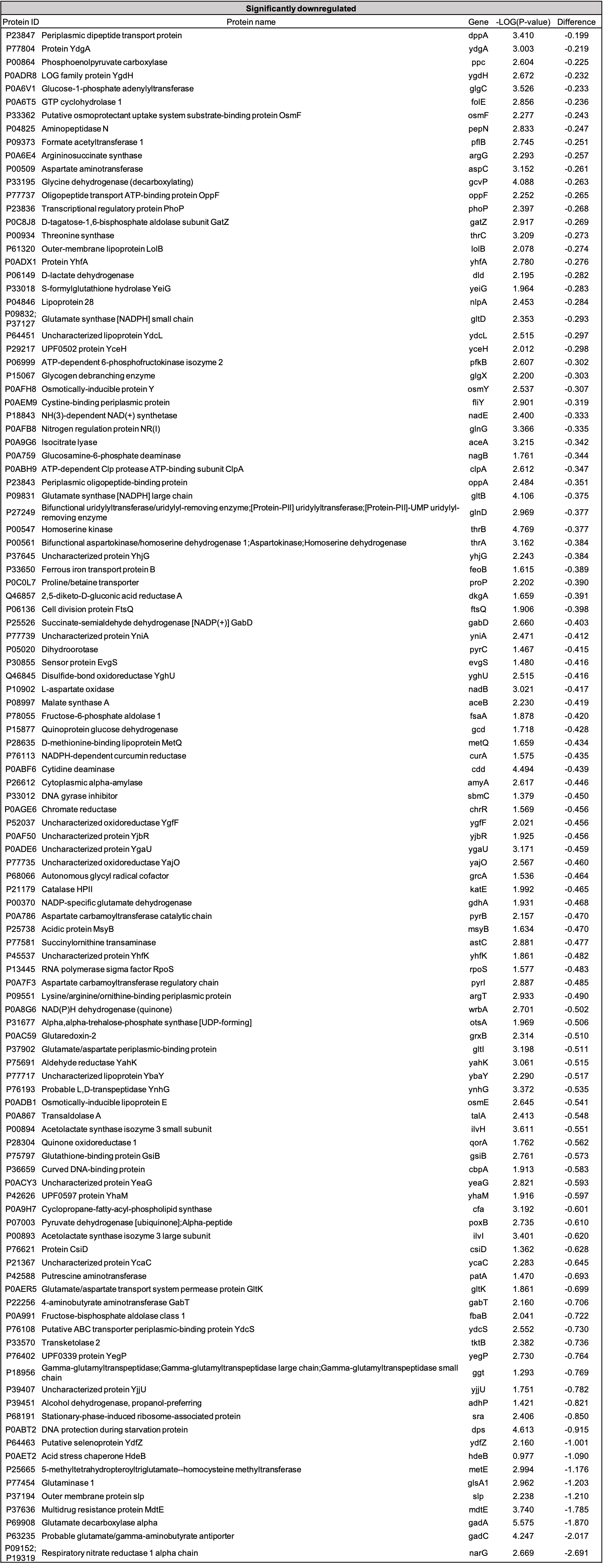
**
