# Supplementary material for: Deacylated tRNA Accumulation Is a Trigger for Bacterial Antibiotic Persistence Independent of the Stringent Response
Source: mBio. 2021 Jun 15;12(3):e01132-21. doi: 10.1128/mBio.01132-21 (PMC8262941; doi:10.1128/mBio.01132-21)
Supplement: TABLE S2 [file mbio.01132-21-st002.docx]

**Table S2. Differentially expressed proteins in *ΔrelA* *pheS* A294S vs. *relA*+ WT *pheS*/*pheT* grown in medium-B.**

**
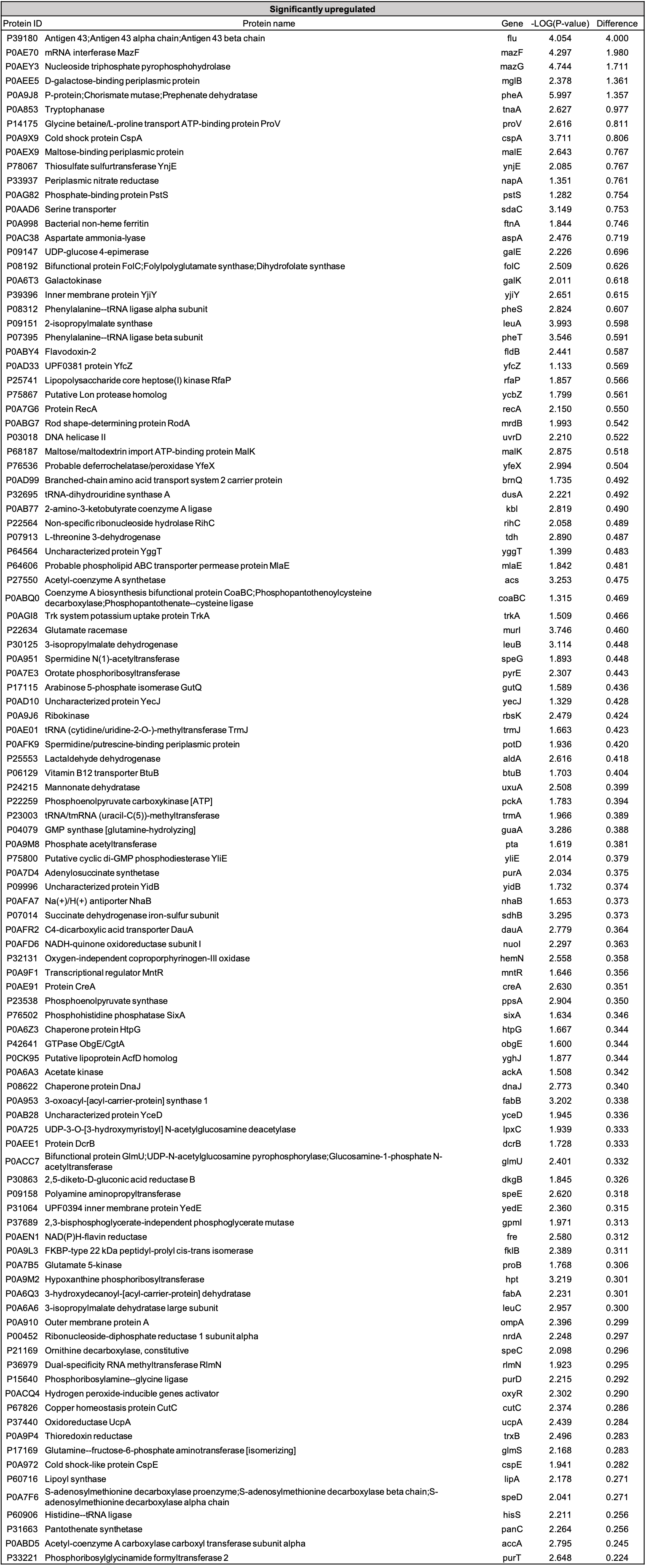

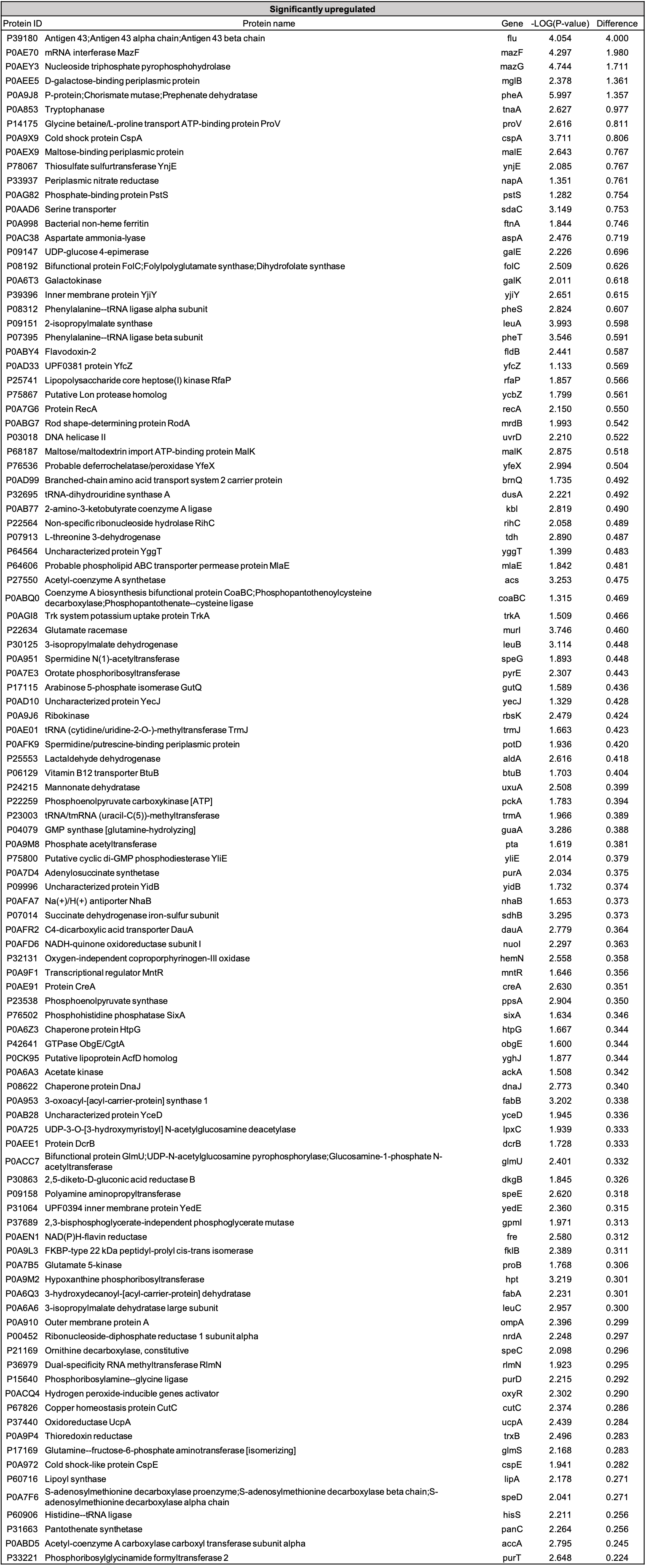
**

**
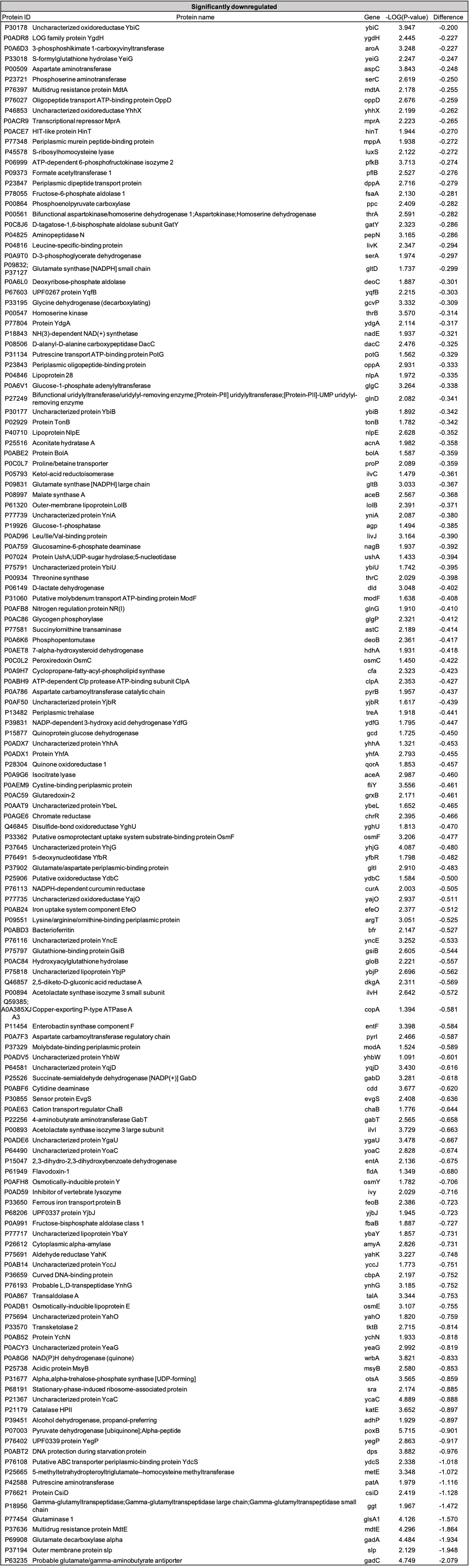

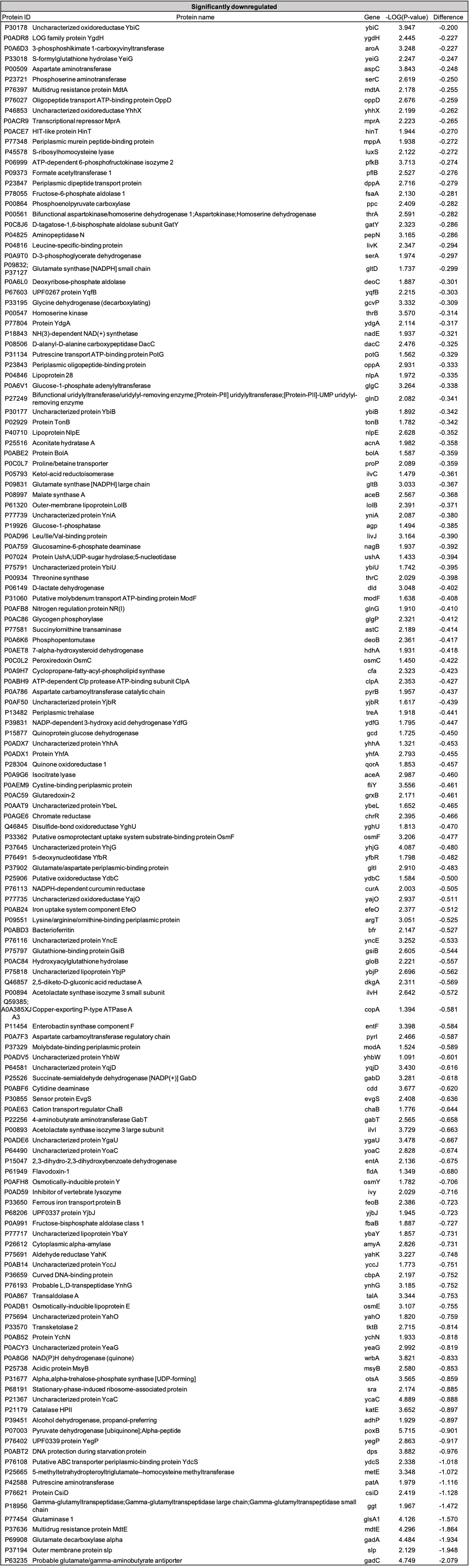
**

**
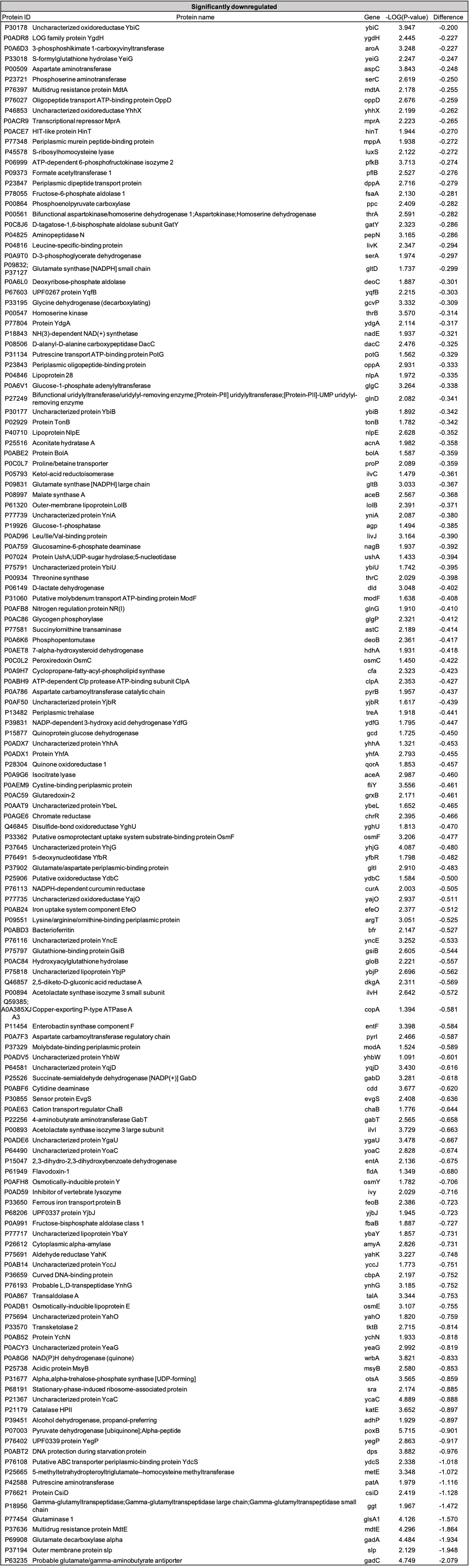
**
