# Supplementary material for: Deacylated tRNA Accumulation Is a Trigger for Bacterial Antibiotic Persistence Independent of the Stringent Response
Source: mBio. 2021 Jun 15;12(3):e01132-21. doi: 10.1128/mBio.01132-21 (PMC8262941; doi:10.1128/mBio.01132-21)

A. Reaction of αA294G PheRS with *m*-Try/Tyr and $tRNA^{Phe}$

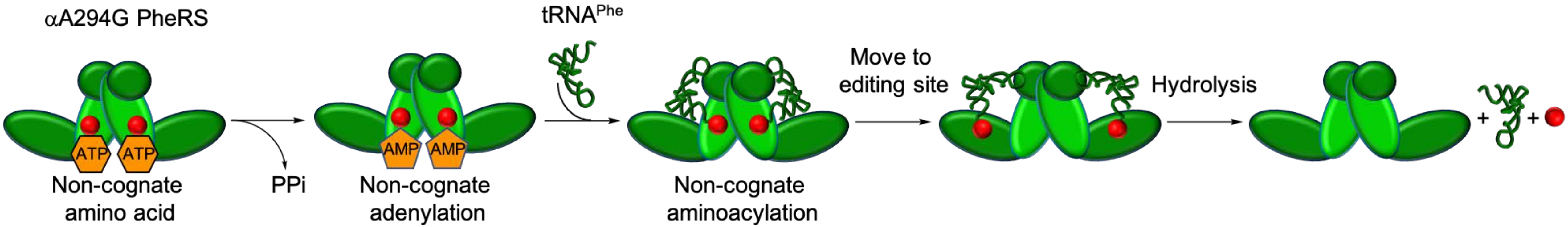

B. Reaction of αA294S PheRS with *m*-Try/Tyr and $tRNA^{Phe}$

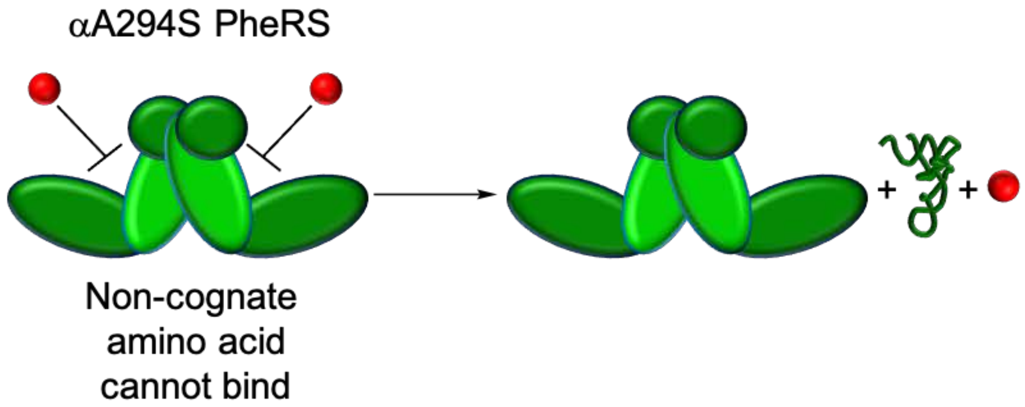

Supplement: FIG S4 [file mbio.01132-21-sf004.pdf]
