# Supplementary material for: Deacylated tRNA Accumulation Is a Trigger for Bacterial Antibiotic Persistence Independent of the Stringent Response
Source: mBio. 2021 Jun 15;12(3):e01132-21. doi: 10.1128/mBio.01132-21 (PMC8262941; doi:10.1128/mBio.01132-21)
Supplement: TEXT S1 [file mbio.01132-21-s0001.docx]

**Supplementary Information Methods and Materials**

**ATP/PPi exchange**

The concentration of active PheRS was determined by active site titrations, as described (1). To determine the amino acid activation kinetics for wild-type and variant PheRS ATP/PPi exchange assays were done as previously described (2). Briefly, reactions were performed at 37 °C in 100 mM Na-Hepes, pH 7.2, 30 mM KCl, 10 mM MgCl_2_, 2 mM NaF, 2 mM ATP, 2 mM ^32^P-PPi (2 cpm/pmol) with various concentrations of phenylalanine (2 – 200 μM), *m*-tyrosine (10 μM – 4 mM), and tyrosine (200 μM – 6 mM), and 25 – 200 nM wild-type PheRS, 25 – 100 nM αA294G PheRS, and 25 nM αA294S PheRS. After 1 – 4 min, 25 μL of the reaction was quenched in a solution containing 1% activated charcoal, 5.6% HClO_4_, and 75 mM PPi. The ^32^P-ATP bound-charcoal was filtered through a 3 mm Whatman disc filter under vacuum and washed 3 times with 5 mL water. The filters were dried, 4 mL of Bio-Safe II Complete Counting Cocktail was added, and the radioactivity was counted by a liquid scintillation counter (Beckman Coulter LS 6500 scintillation system).

**Quantification of aminoacylated and deacylated tRNA**

Total RNA was purified from *E. coli* cells under acid conditions and on ice to avoid deacylation of the aminoacylated tRNA as described (3, 4). Cultures (20 mL in either mediu -A or medium-B) were grown to an OD_600_ of 1.0 and then harvested by centrifugation at 4,500 x *g* for 10 min at 4 °C. The cell pellet was suspended in 400 μL cold lysis buffer (0.3 M sodium acetate, pH 4.5, and 10 mM EDTA). An equal volume of phenol/chloroform, pH 4.5 (1:1) was added to the cell suspension and vortexed for 30 s and then rested on ice for 30 s, repeating three times followed by centrifugation at 18,600 x *g* for 15 min at 4 °C. The aqueous layer was removed, and the phenol/chloroform extraction was repeated. The final aqueous layer was removed and 2.7 volumes of cold 100% ethanol was added to precipitate the RNA at -20 °C for 1 h. The RNA was pelleted by centrifugation at 18,600 x *g* for 30 min at 4 °C. The RNA pellet was suspended in 200 μL of cold lysis buffer and 2.7 volumes of cold 100% ethanol was added to the RNA suspension and was precipitated and pelleted as previously described. After centrifugation, the final RNA pellet was suspended in 40 μL of 0.3 M sodium acetate, pH 4.5, and 1 mM EDTA and stored at -80 °C for up to two weeks.

Acid/urea gel electrophoresis and northern blotting were performed as described (5). Briefly, loading dye (7 M urea, 0.3 M sodium acetate, pH 5.2, 0.5 μg/mL bromophenol blue) was added to 10 μg of purified aminoacylated-tRNA and deacylated-tRNA control (purified aminoacylated-tRNA incubated at 42 °C in Tris-HCl, pH 9.0, for 1 h). Samples were separated on a 14% polyacrylamide, 0.3 M sodium acetate, pH 5.2, 7 M urea gel (61 mm x 82 mm) in a Bio-Rad Mini Protean 3 apparatus. The gel was run at 50 volts for 27 h at 4 °C in 0.3 M sodium acetate, pH 5.2. The running buffer was changed every 7-8 h to maintain an acidic pH. Electrophoretic transfer of the separated RNA onto a Bio-Rad Zeta-probe cationized nylon membrane was performed in transfer buffer (10 mM tris-acetate, pH 7.8, 5 mM sodium acetate, 0.5 mM EDTA) at 44 volts for 2 h at 4 °C in a Bio-Rad Criterion blotter. Gel, membrane, and filter papers were soaked in transfer buffer for 10 min prior to electrophoretic transfer. After electrophoretic transfer the RNA was UV-crosslinked to the membrane followed by pre-hybridization (5X saline sodium citrate buffer, 20 mM Na_2_HPO_4_, pH 7.2, 7% sodium dodecyl sulfate, 1X Denhardt’s solution, 100 mg/mL denatured salmon sperm DNA) for 1 h at 50 °C and then hybridization with ^32^P-5’-end labeled probe (5’-aatcgaaccaaggacacgggg-3’) overnight at 50 °C (6). The probe was ^32^P-5’-end labeled using γ-^32^P-ATP and T4 polynucleotide kinase (New England BioLabs). Finally, the blot was washed according to the manufacturers protocol (Bio-Rad Laboratories). Visualization and quantification were performed by phosphor imaging.

**Targeted metabolomic analysis and quantification**

Cultures (5 mL in either medium-A or medium-B) were grown until OD_600_ of 0.3 and were then harvested by vacuum-filtration onto a nylon membrane (Whatman, 0.45 μM). The cells were washed with 3 mL of cold-water three times and the filter was removed from the vacuum filter support and submerged in 800 μL of -20 °C 40:40:20 acetonitrile/methanol/water in Eppendorf tubes. At this point, 10 ppm of ^13^C-ATP/^13^C-GTP mass spectrometer (MS) internal standard (IS) and 1X Halt protease and phosphatase inhibitor cocktail (ThermoFisher Scientific) was added to the extraction mixture and incubated at -20 °C for 15 min. The filter was removed and washed with 200 μL of extraction solution and centrifuged at 16,000 x *g* for 5 min at 4 °C. The metabolite extract was transferred to a new Eppendorf tube and the cell pellet was suspended in 100 μL of extraction solution and incubated on ice for 15 min then centrifuged as previously described. The supernatant was added to the previous supernatant and dried via a speed vac at room temperature overnight for liquid chromatography (LC)/MS analysis.

**References**

1. Ibba M, Kast P, Hennecke H. 1994. Substrate specificity is determined by amino acid binding pocket size in Escherichia coli phenylalanyl-tRNA synthetase. Biochemistry 33:7107-12.

2. Roy H, Ling J, Alfonzo J, Ibba M. 2005. Loss of editing activity during the evolution of mitochondrial phenylalanyl-tRNA synthetase. J Biol Chem 280:38186-92.

3. Zaborske J, Pan T. 2010. Genome-wide analysis of aminoacylation (charging) levels of tRNA using microarrays. J Vis Exp doi:10.3791/2007.

4. Mohler K, Mann R, Ibba M. 2017. Isoacceptor specific characterization of tRNA aminoacylation and misacylation in vivo. Methods 113:127-131.

5. Jester BC, Levengood JD, Roy H, Ibba M, Devine KM. 2003. Nonorthologous replacement of lysyl-tRNA synthetase prevents addition of lysine analogues to the genetic code. Proc Natl Acad Sci U S A 100:14351-6.

6. Bullwinkle TJ, Ibba M. 2016. Translation quality control is critical for bacterial responses to amino acid stress. Proc Natl Acad Sci U S A 113:2252-2257.
